# YOLOv8-Peas: a lightweight drought tolerance method for peas based on seed germination vigor

**DOI:** 10.3389/fpls.2023.1257947

**Published:** 2023-09-28

**Authors:** Haoyu Jiang, Fei Hu, Xiuqing Fu, Cairong Chen, Chen Wang, Luxu Tian, Yuran Shi

**Affiliations:** ^1^College of Artificial Intelligence, Nanjing Agricultural University, Nanjing, China; ^2^College of Engineering, Nanjing Agricultural University, Nanjing, China; ^3^College of Information Management, Nanjing Agricultural University, Nanjing, China

**Keywords:** pea seed, seed vitality, drought tolerance, lightweight, YOLOv8

## Abstract

**Introduction:**

Drought stress has become an important factor affecting global food production. Screening and breeding new varieties of peas (Pisum sativum L.) for drought-tolerant is of critical importance to ensure sustainable agricultural production and global food security. Germination rate and germination index are important indicators of seed germination vigor, and the level of germination vigor of pea seeds directly affects their yield and quality. The traditional manual germination detection can hardly meet the demand of full-time sequence nondestructive detection. We propose YOLOv8-Peas, an improved YOLOv8-n based method for the detection of pea germination vigor.

**Methods:**

We constructed a pea germination dataset and used multiple data augmentation methods to improve the robustness of the model in real-world scenarios. By introducing the C2f-Ghost structure and depth-separable convolution, the model computational complexity is reduced and the model size is compressed. In addition, the original detector head is replaced by the self-designed PDetect detector head, which significantly improves the computational efficiency of the model. The Coordinate Attention (CA) mechanism is added to the backbone network to enhance the model's ability to localize and extract features from critical regions. The neck used a lightweight Content-Aware ReAssembly of FEatures (CARAFE) upsampling operator to capture and retain detailed features at low levels. The Adam optimizer is used to improve the model's learning ability in complex parameter spaces, thus improving the model's detection performance.

**Results:**

The experimental results showed that the Params, FLOPs, and Weight Size of YOLOv8-Peas were 1.17M, 3.2G, and 2.7MB, respectively, which decreased by 61.2%, 61%, and 56.5% compared with the original YOLOv8-n. The mAP of YOLOv8-Peas was on par with that of YOLOv8-n, reaching 98.7%, and achieved a detection speed of 116.2FPS. We used PEG6000 to simulate different drought environments and YOLOv8-Peas to analyze and quantify the germination vigor of different genotypes of peas, and screened for the best drought-resistant pea varieties.

**Discussion:**

Our model effectively reduces deployment costs, improves detection efficiency, and provides a scientific theoretical basis for drought-resistant genotype screening in pea.

## 1 Introduction

Peas (*Pisum sativum* L.), one of the four major global legume crops in the world, are rich in protein, fiber, and other key nutrients, providing an important food source for humans and animals(Cousin, 1997; Dahl et al., 2012). Their rich dietary fiber and antioxidant contents and important biomolecules have significant potential in promoting health and preventing chronic diseases (Devi et al., 2023). With global climate change, water scarcity has become the major abiotic factor limiting the survival and output of peas, so screening and breeding new drought-tolerant pea varieties is the key to enhance their yield and quality (Magyar-Tábori et al., 2011). Seed germination rate and germination index can be calculated by examining the germination status of pea seeds, which are key indicators for assessing the vigor of seed germination, reflecting the ability of seeds to germinate in suboptimal environments such as low moisture availability (Ranal and Santana, 2006). However, traditional methods of seed germination detection usually destroy seed integrity and the processes are labor intensive, time consuming, and rely on subjective judgment, limiting throughput and accuracy (Mir et al., 2015; Jahnke et al., 2016). Therefore, a high-precision, automated, and high-throughput assay should be proposed for pea seed germination vigor monitoring.

With the development of machine learning, many scholars started to explore the nondestructive testing of agricultural seed viability (Mladenov et al., 2019; Tang et al., 2020). For example, He et al. (2019) achieved the detection of rice seed germination vigor in different years by near-infrared hyperspectral imaging (NIR-HSI) technique combined with an Extreme Learning Machine model with 93.67% accuracy. Wang et al. (2022b) used Support Vector Machine (SVM) method to achieve rapid detection of seed vigor of glutinous maize under different aging levels. de Medeiros et al. (2020a) proposed a seed quality classification method that uses Fourier transform near infrared (FT-NIR) spectroscopy and X-ray imaging techniques to merge data and compare and analyze the detection performance of six classification models. de Medeiros et al. (2020b) combined automated X-ray analysis and Latent Dirichlet Allocation (LDA) models to predict the germination rate and seedling vigor of Cyathea seeds. Škrubej et al. (2015) used a Artificial Neural Network (ANN) to achieve accurate assessment of tomato seed germination. However, machine learning mainly relies on low-level features, making it difficult to extract deep semantic information. Especially in recognizing the sprouting process, changes in the light source and acquisition environment may lead to degradation of image quality, and the complex morphology of the root system may produce confusing and blurred areas in the image (Barredo Arrieta et al., 2020; Fu et al., 2021). The above problem poses a challenge to the use of machine learning for seed germination detection as it requires specific algorithms to be developed for different environments with low robustness. And deep learning models such as Convolutional Neural Networks (CNN) can capture different levels of features of an image. Such a hierarchical structure, from the edges and textures at the bottom to the overall morphology and structure at the top, allows deep learning to capture richer and more complex semantic information. This enables these complexities and disturbances to be more accurately identified and dealt with, improving the quality of image interpretation and analysis.

With the rapid development of deep learning, target detection technology based on deep learning has become an important driver for the transformation of agricultural production to digitalization and intelligence (Girshick et al., 2014; Kamilaris and Prenafeta-Boldú, 2018). In particular, the You Only Look Once (YOLO) family of algorithms, with its excellent detection speed and accuracy, has become the most widely used and efficient target detection methods in the field (Redmon et al., 2016; Redmon and Farhadi, 2018; Bochkovskiy et al., 2020; Jiang et al., 2022; Li et al., 2022; Wang et al., 2023). Many scholars have optimized and improved it according to specific problems in agricultural production to further improve detection accuracy and efficiency (Wu et al., 2020). For example, Zhao et al. (2023) designed a YOLO-r network to assess the germination status and total number of germinated rice seeds. Kundu et al. (2021) used an improved YOLOv5 network for seed classification and quality detection to address the challenge of differentiating seeds of different crops in mixed cropping. Zhang and Li (2022) constructed a multi-growth detection model for hydroponic oilseed rape based on YOLOv5, which has helped to accurately monitor crop survival and significantly improve space utilization in cultivation scenarios such as greenhouses. Fu et al. (2022) produced a YOLOv4-based wheat salt tolerance detection platform with 97.59% detection accuracy for wheat salt tolerance genotype screening. Wang et al. (2022a) combined transformer with the YOLOv5 backbone to enhance the sensitivity of the model to weeds and added spatially adaptive feature fusion to reduce the loss of features. Zhang et al. (2022) used YOLOv5-s to detect dragon fruit in different environments and added a coordinate attention mechanism to enable the accuracy of model localization and the ability to identify dense targets, as well as introduced SIOU to accelerate model convergence (Gevorgyan, 2022).

The above research results show that the target detection technology has a wide range of applications in the field of agriculture and achieved better results, based on the combination of agricultural production and target detection of smart agriculture has become a major trend in the development of modern agriculture in various countries. YOLOv8 is the latest proposed target detection model, which achieves the best level in several indexes such as detection accuracy and real-time performance. However, there are still some limitations in pea germination vigor detection. First of all, uneven illumination can lead to imbalance in image color and contrast, and due to the large seed particles, it can lead to severe root overlap and affect the image quality. To cope with the impact of these complex conditions on model performance, the model needs to be provided with a large amount of training data from different environments, whereas pea germination is a long process and difficult to collect a large amount of data in a given environment, and it takes a lot of time to collect and label the data. Data augmentation is a better option for boosting the amount of data in a given environment. Although some data enhancements such as mosaic enhancement are used in YOLOv8, it is difficult to satisfy the model’s need for specific data due to its inability to scale the amount of data. And many data enhancement methods cannot be expanded together with the labeled data due to its complexity, and need to be labeled again, which is very consuming of manpower and time costs. Second, although the buds are a small percentage of the whole pea, they are a key basis for determining whether or not to sprout, and each bud needs to be matched to the coordinates of the corresponding pea. YOLOv8, on the other hand, has a weak feature extraction capability for small targets and can further affect the model’s ability to capture and localize key features due to issues such as root-bud interleaving. At the same time, the quality of up-sampling to generate feature maps is also the key to the accuracy of the model. YOLOv8 uses a simple bilinear interpolation method for up-sampling, which has some limitations in processing the semantic information of the feature maps and the perceptual range. In addition, from an economic cost point of view, practical models should be lightweight while maintaining accuracy to accommodate low-performance device deployments. To address these issues, this paper presents a lightweight target detection model YOLOv8-Peas specifically for pea germination vigor detection. The main contributions are as follows:

(1) Enhanced data augmentation strategy: In order to improve the robustness of the model in real-world application scenarios, we adopt a variety of simple but effective data augmentation techniques to expand the image and label data correspondingly. This not only reduces the labor cost, but also improves the model’s ability to adapt to different environments and changes, which further ensures the accuracy of detection. (2) Lightweight characteristics: The YOLOv8-Peas model proposed in this paper has been deeply lightweighted and designed to successfully reduce the number of model parameters and computation by using deeply differentiable convolution, C2f-Ghost structure and specially designed PDetect detection head. This ensures model deployment and detection in resource-limited environments. (3) Introduction of the Coordinate Attention Mechanism: In order to capture the germination features of peas more accurately in complex backgrounds and environments, we introduced the Coordinate Attention Mechanism for the first time in this type of task. This mechanism enables the model to pay more targeted attention to key germination regions in the image. (4)Optimization of the up-sampling operator: CARAFE is able to adaptively generate up-sampling kernels and perform instance-specific content-aware processing, which effectively integrates a larger range of contextual information, and is able to accurately recover image details without the need to introduce more additional learning parameters. (5) Verification of practical application scenarios: This paper also verified the practical application effect of the model in the drought environment simulated by using PEG6000, collected the growth images of four genotypes of pea seeds under two kinds of drought conditions, and evaluated the germination rate and germination index with the help of the optimized model to achieve the screening of drought-resistant genotypes of peas, which further demonstrated the value of its practical application.

## 2 Materials and methods

### 2.1 Data acquisition equipment

**Figure 1:**
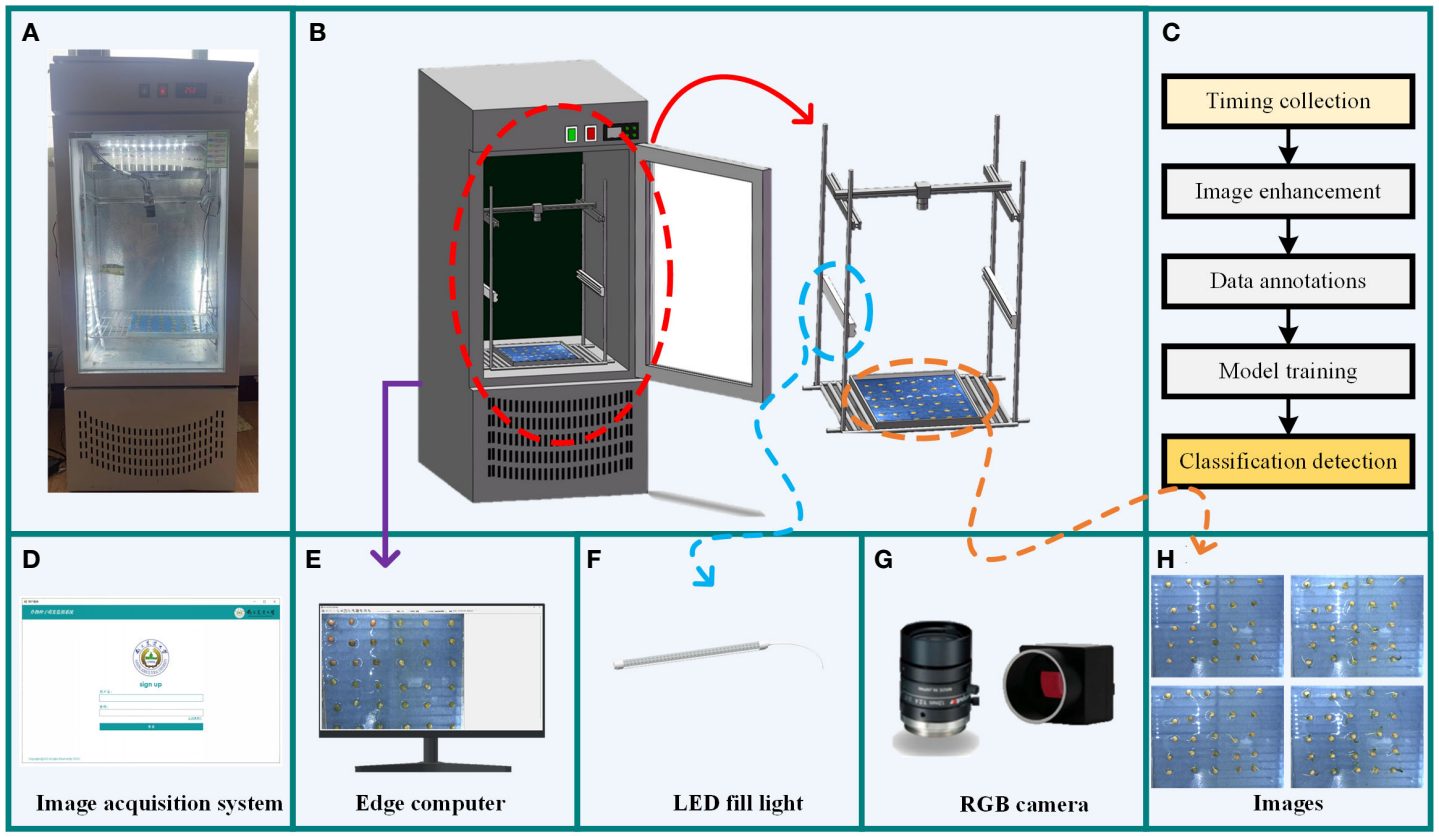
Pea seed germination data acquisition equipment. **(A)** Physical image of the incubator. **(B)** Structure of the incubator. **(C)** Module training process. **(D)** Data acquisition system. **(E)** Edge computer. **(F)** LED fill light. **(G)** Data acquisition camera. **(H)** Schematic of the acquired images.

**Figure 1** depicts the structure of the seed germination incubator and image acquisition system used for our experiments. The incubator is equipped with an embedded PTC hot air circulation system and LED lighting with a temperature range of 5°C to 50°C to provide a constant and suitable environment to promote seed germination. Mounted on top of the incubator is an MV-HS510GC model Vivision RGB industrial camera, which utilizes a GigE Gigabit network high-speed interface for data transmission and has the advantage of being small and cost-effective. The lens is a Comptroller M1224-MPW2 model, connected to the camera via the C-mount interface, with an image capture resolution of 2448×2048 pixels. The camera unit was selected in 12 mm fixed focus mode and mounted 40 cm directly above the subject. It can collect high-quality image data of seeds at each germination stage in real time, which provides the necessary data support for model training. The crop seed germination collection system allows easy operation of the imaging equipment and storage of data. Users can select the shooting interval and adjust parameters such as contrast and screen size to obtain high-quality images through the software interface on the PC. The acquired images are stored, and image processing is performed. Finally, the processed dataset is labeled, and a pea sprouting detection model is trained.

### 2.2 Data acquisition and pre-processing

#### 2.2.1 Data acquisition

**Figure 2:**
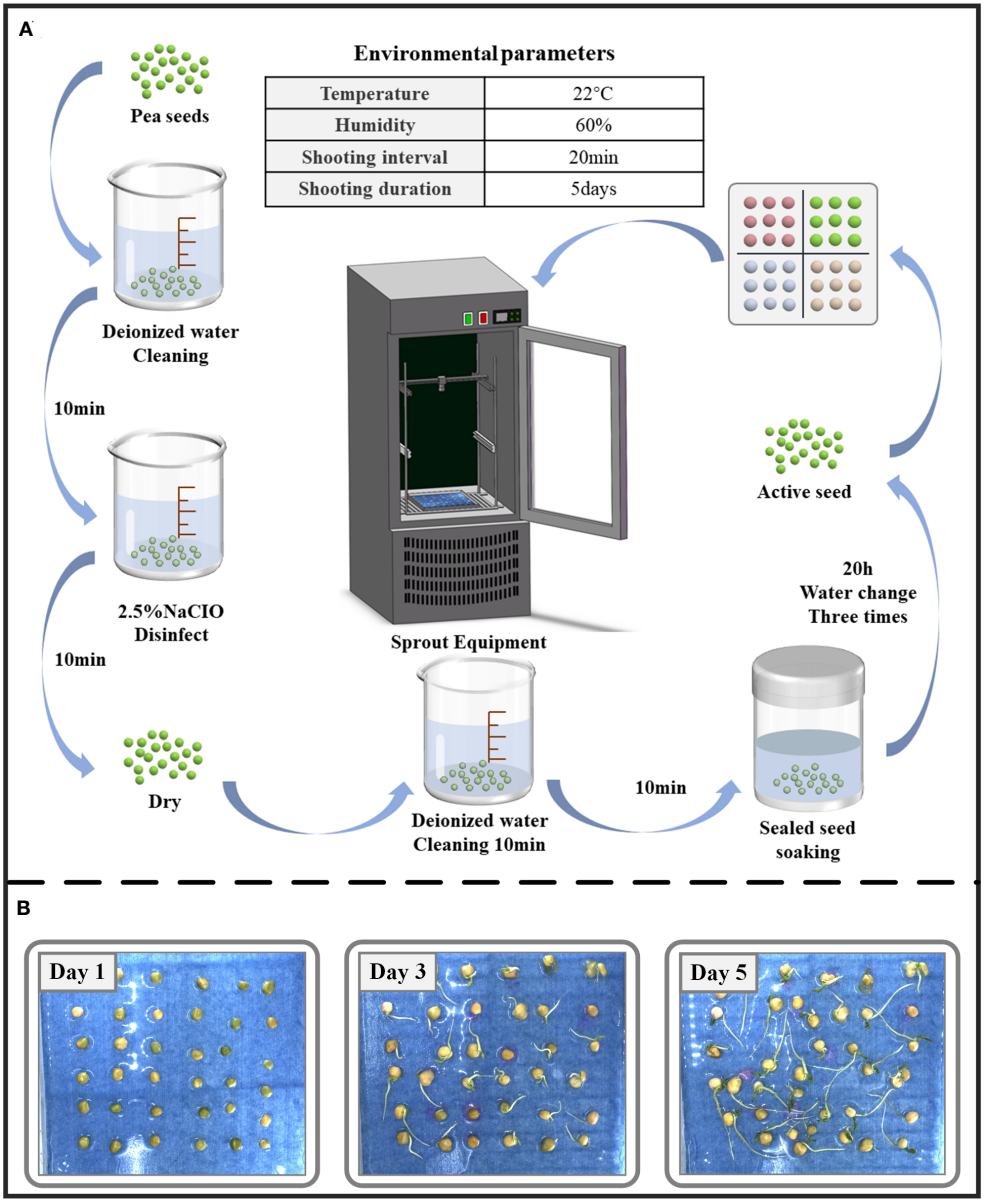
**(A)** Experimental flow chart. **(B)** Schematic of pea seed growth process.

A total of 126 uniform and full pea seeds were selected for the seed germination experiment, and the experimental procedure is shown in **Figure 2A**. Each experiment lasted for 5 days, and the experiment was repeated four times. After screening, a total of 1017 images were collected throughout the experimental period, and all images were saved in.jpg format with a resolution of 2448×2048 pixels. The growth process of pea seeds is shown in **Figure 2B**. At the same time, drought stress experiments were conducted. Different drought conditions were simulated using different concentrations of PEG6000 solution to inhibit water uptake in pea. Zhonghua No. 6, Zhonghua No. 11, Qizhen No. 76, and Gancui No. 2, four pea varieties with different drought tolerance were selected for the experiment, and 36 seeds were placed in each culture plate, including four pea varieties with nine seeds each. A single experiment lasted five days. Two sets of replicated trials were set up for each variety to minimize errors. All culture plates were subjected to two treatments in the same environment: a control with deionized water added (CK) and a 10% concentration of PEG6000 solution to simulate drought stress (S1). A total of 1440 images were collected during the experiment.

#### 2.2.2 Data pre-processing

**Figure 3:**
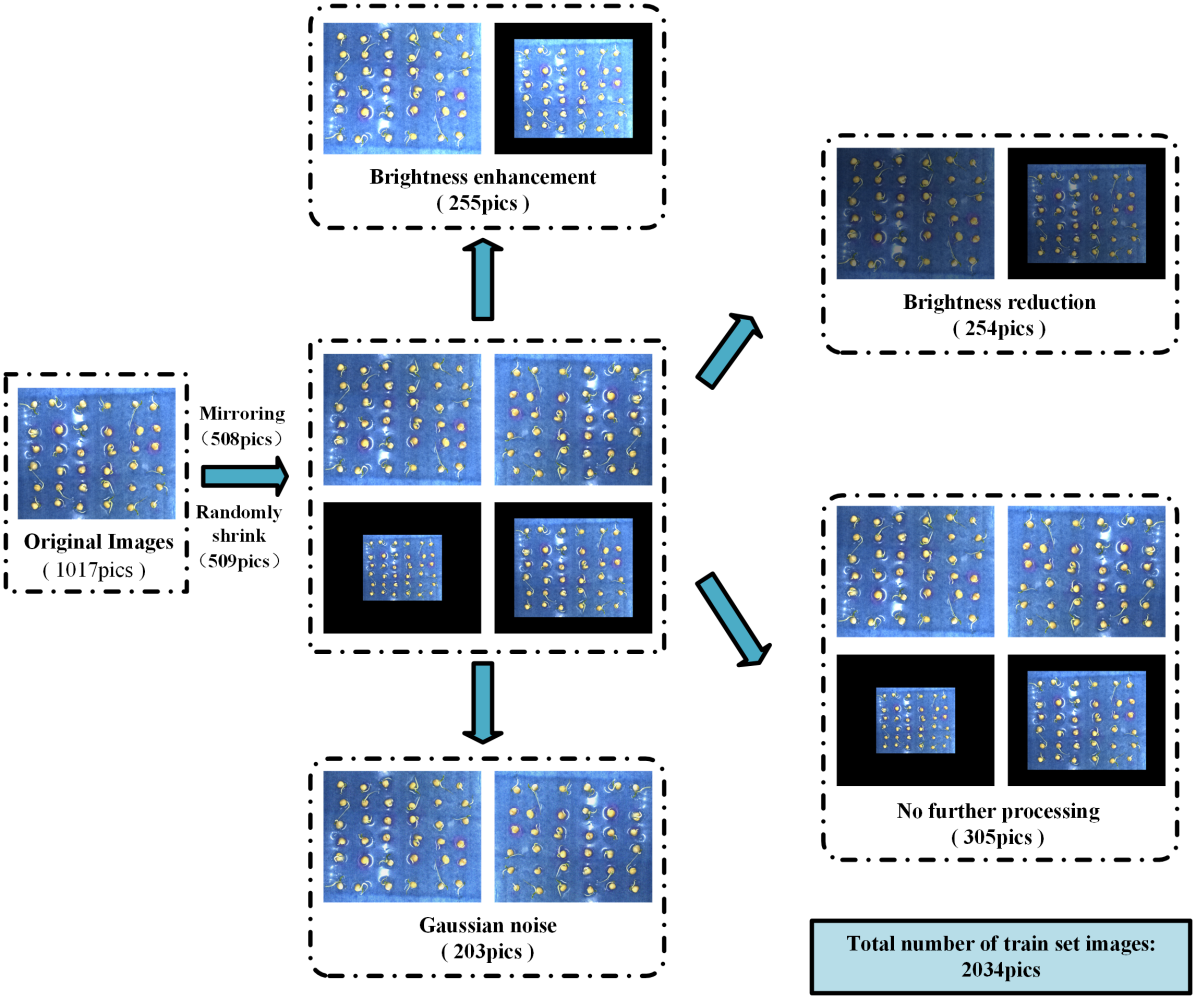
Data augmentation process.

The main factors affecting the accuracy of pea germination detection are picture brightness, seed placement, the number of seeds in a single picture, root interlacing, etc., and considering the cost of manpower annotation, in order to effectively expand the amount of data under different conditions and increase the robustness of the proposed method. We consider using the following simple and effective enhancement methods to enhance the corresponding image and label data: 1) Enhance the recognition ability of the model under different lighting conditions by adjusting the image brightness to cope with brightness fluctuations in practical applications. 2) Picture mirroring simulates different orientations of pea growth to enhance detection. 3) Stochastic scaling improves the model’s ability to recognize at different scales. 4) Add Gaussian noise to simulate various perturbations encountered by images in the real world (e.g., sensor noise due to poor lighting conditions or high temperatures), thus enhancing the model’s resistance to noise interference. Equation (1) represents the original image Pi obtained by data enhancement ${ℱ}_{l}$ to obtain Pi′ , where *l* represents the method of data enhancement. In our study, each image was subjected to at least one data enhancement technique. This composite data enhancement strategy aims to enhance the model’s recognition performance in complex environments and reduce the interference of external factors on detection, thus enhancing the model’s generalization and effectively preventing overfitting phenomena. The specific data enhancement process is shown in **Figure 3**. Our final dataset contained 2034 images, 1017 original images, and 1017 images after data enhancement.

The correspondence between the enhanced label coordinates and the original label coordinates is shown in Equations (2) and (3). The label corresponding to each image Pi is Li, with n annotation boxes inside Li. The coordinates of the upper left corner of the k-th (k< n) annotation box are ( ${\text{X}}_{\text{k}1},{\text{Y}}_{\text{k}1}$ ), and the coordinates of the lower right corner are ( ${\text{X}}_{\text{k}2},{\text{Y}}_{\text{k}2}$ ). The label ${\text{Li}}^{\prime }$ corresponding to Pi′ can be calculated based on the label Li of Pi combined with the specific enhancement method. ℳ  represents the mapping relationship of the labeled data. $\text{L}{{\text{i}}^{\prime }}_{\text{k}}$ denotes the coordinates of the upper-left and lower-right corners of the k-th labeled box of ${\text{Li}}^{\prime }$ . W and H represent the image width and height, respectively, and s represents the scaling factor (0< s< 1 for scaling down, s > 1 for scaling up). Since adding Gaussian noise results in some perturbation of the coordinates, we assume that the perturbations are δx and δy .


(1) $$\text{P}{\text{i}}^{\prime }={\text{ℱ}}_{\text{l}}\left(\text{Pi}\right)$$

(2) $$\text{L}i_{k}^{\prime }={ℳ}_{l}\left(\text{L}i_{k}\right)=\{\begin{array}{ll} \left(X_{k1},Y_{k1}\right),\left(X_{k2},Y_{k2}\right) & \text{l}=\text{brightness adjustment}\\ \left(\text{W}-X_{k2},Y_{k1}\right),\left(\text{W}-X_{k1},Y_{k2}\right) & \text{l}=\text{Horizontal Flip}\\ \left(X_{k1},\text{H}-Y_{k2}\right),\left(X_{k2},\text{H}-Y_{k1}\right) & \;l=\text{vertical flip}\\ \left(\text{s}\times X_{k1},\text{s}\times Y_{k1}\right),\left(\text{s}\times X_{k2},\text{s}\times Y_{k2}\right) & \;\text{l}=\text{random scaling}\\ \left(X_{k1}+\text{δx},Y_{k1}+\text{δy}\;\right),\left(X_{k2}+\text{δx}\;,Y_{k2}+\text{δy}\right) & \;\;\text{l}=\text{Gaussian noise} \end{array}\;$$

(3) $$\text{L}i^{\prime }=\sum_{k=1}^{n}\text{L}i_{k}^{\prime }$$

**Figure 4:**
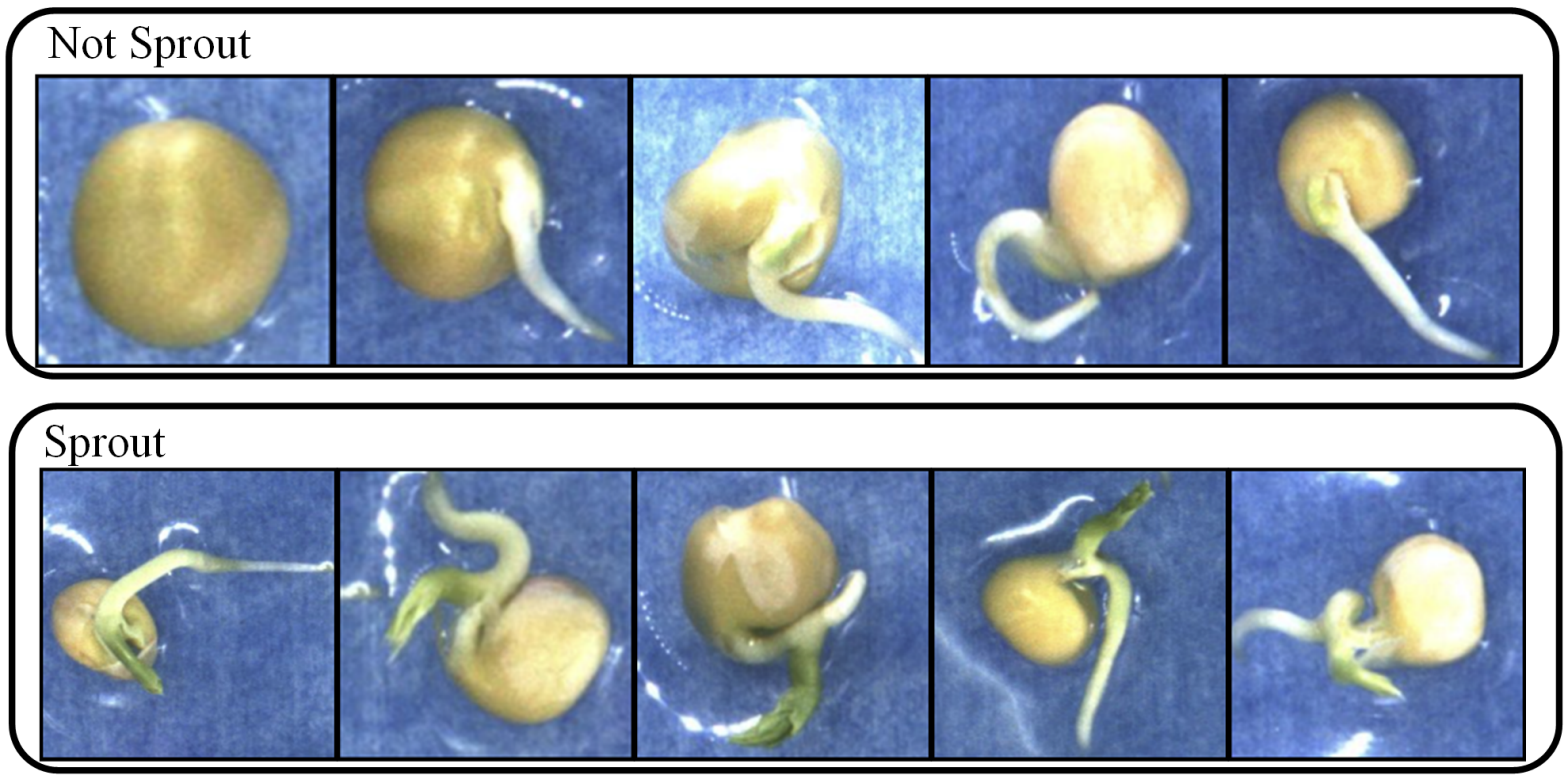
Schematic of not sprout and sprout pea seeds.

The LabelImg tool was used to annotate the dataset, and evaluation criteria were developed for the germination status of the seeds. We label a seed as germinated when the length of the germ reaches half the length of the seed itself (the length of the germ to the bottom of its seed). An example is shown in **Figure 4**.

### 2.3 Design for YOLOv8-Peas

**Figure 5:**
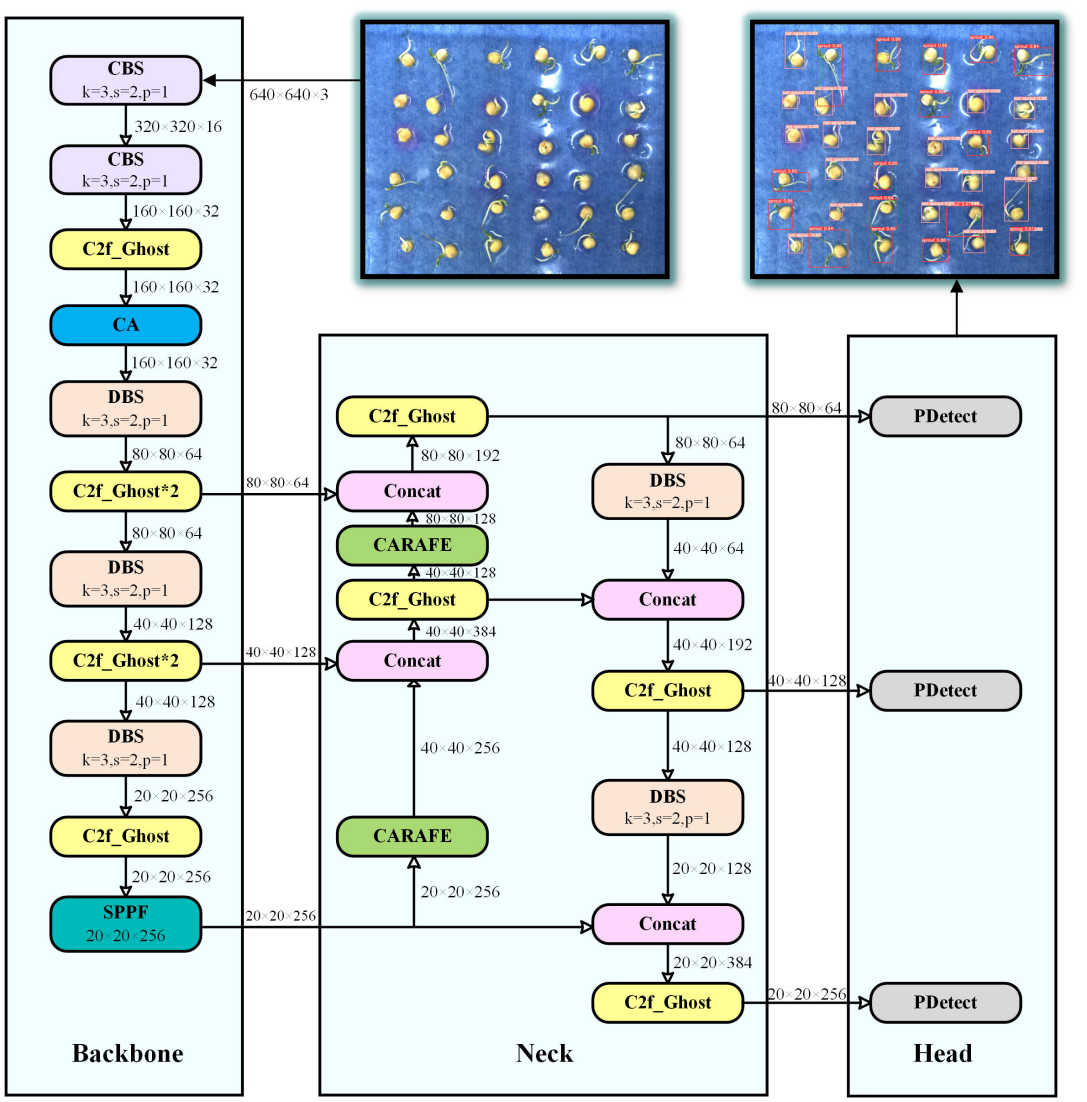
YOLOv8-Peas detector structure scheme. The k in the module represents the convolution kernel size, s represents the step size, and p represents the pooling kernel size.

To improve the accuracy of pea germination detection and to achieve model lightweighting to reduce the deployment cost. We propose an efficient detection model YOLOv8-Peas based on YOLOv8-n. As shown in **Figure 5**, this model included three major parts: the main stem, the neck, and the predicted head. The Spatial Pyramid Pooling - Fast (SPPF) module in the backbone section can handle objects at different scales, and it has a set of maximal pooling layers with different sized pooling kernels, which allows the network to extract features at multiple scales and enhances the model’s adaptability to the target scale. In the neck of the model, a Path Aggregation Network for Feature Pyramid Network (PAN-FPN) structure is used for feature fusion to deliver deep semantic features in a top-down manner. In the detection head section, decoupled heads are used to enhance the classification and localization capabilities of the model.

Compared to YOLOv8, we have made the following optimizations: 1) In the C2f module, we adopt the efficient Ghost_Bottleneck structure to optimize Bottleneck, which not only improves the performance of the model, but also reduces the computational complexity of the model, making the model more lightweight and efficient. 2) Some CBS modules (consisting of convolutional layers, a batch normalization layer, and a SiLU activation function) have been replaced with DBS modules with fewer parameters and more efficient calculations (consisting of depthwise convolutional layers, a batch normalization layer, and a SiLU activation function). 3) We designed the PDetect detection head using the PCC module, which effectively reduces redundant calculations and memory access, thereby improving the detection rate and significantly reducing the number of parameters. 4) At the backbone, we introduced the CA mechanism, which helps the model to extract and localize the germination features of peas more accurately, while reducing the interference of complex background. 5) At the neck, we adopted the CARAFE upsampling operator, a lightweight upsampling strategy that enhances the model’s perception of details and optimizes the representation of semantic features.

#### 2.3.1 Lightweight design

In this section, we detail the lightweight design strategy employed in YOLOv8-Peas. In order to achieve more efficient and accurate object detection, we have introduced several new modules and technologies. These innovative designs aim to reduce the computational complexity and volume of the model, thereby meeting the requirements of embedded devices and real-time applications while maintaining high detection accuracy. The following subsections describe in detail the design and implementation of the C2f-Ghost module, the DBS module and the PDetect detection header in turn.

##### 2.3.1.1 C2f-Ghost

**Figure 6:**
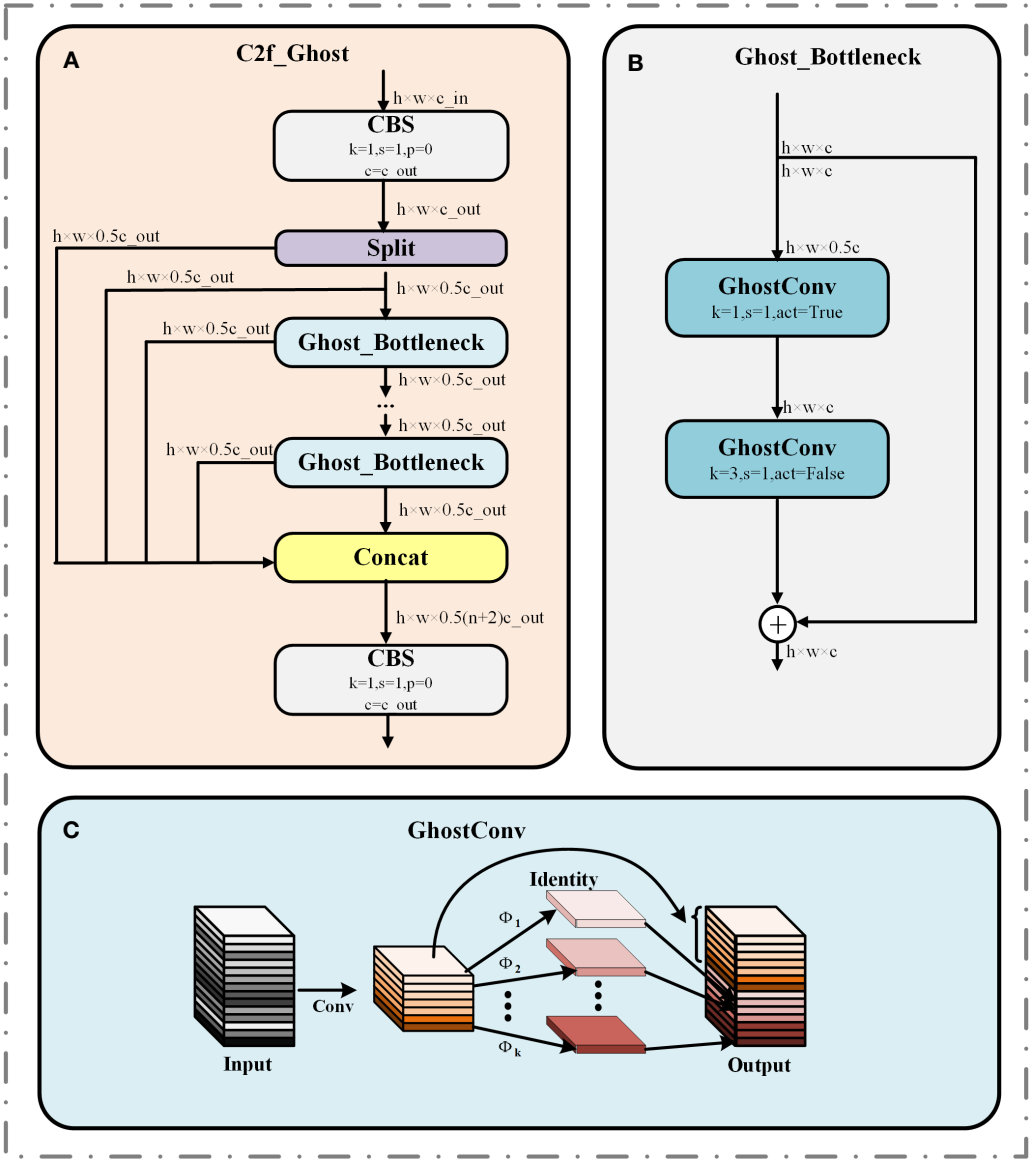
Structure diagram of C2f_Ghost in YOLOv8-Peas. **(A)** C2f_Ghost module. **(B)** Ghost_Bottleneck module. **(C)** GhostConv module.

To compress model size and reduce model deployment costs. We will replace the Bottleneck inside the C2f module with Ghost_ Bottleneck, which in turn forms the C2f Ghost module (Han et al., 2020). The structures of C2f-Ghost and Ghost_Bottleneck are demonstrated in **Figures 6A, B**, where c is the number of channels and the activation function is used when act equals True.

Ghost_Bottleneck is an efficient network structure based on the GhostConv module, and the GhostConv structure is shown in **Figure 6C**. GhostConv obtains a feature map comparable with the standard convolution through a two-stage process, which significantly reduces the operational parameters and achieves the dual optimization of computational efficiency and model accuracy. First, the initial convolution operation is performed with a small computational load, and the remaining feature maps are generated by inexpensive linear transformations ${\text{Φ}}_{\text{k}}$ . Finally, the feature maps generated twice are superimposed on the channel dimensions to generate the final output. This design approach improves the efficiency of the model while maintaining a rich feature representation capability.

##### 2.3.1.2 DBS module

**Figure 7:**
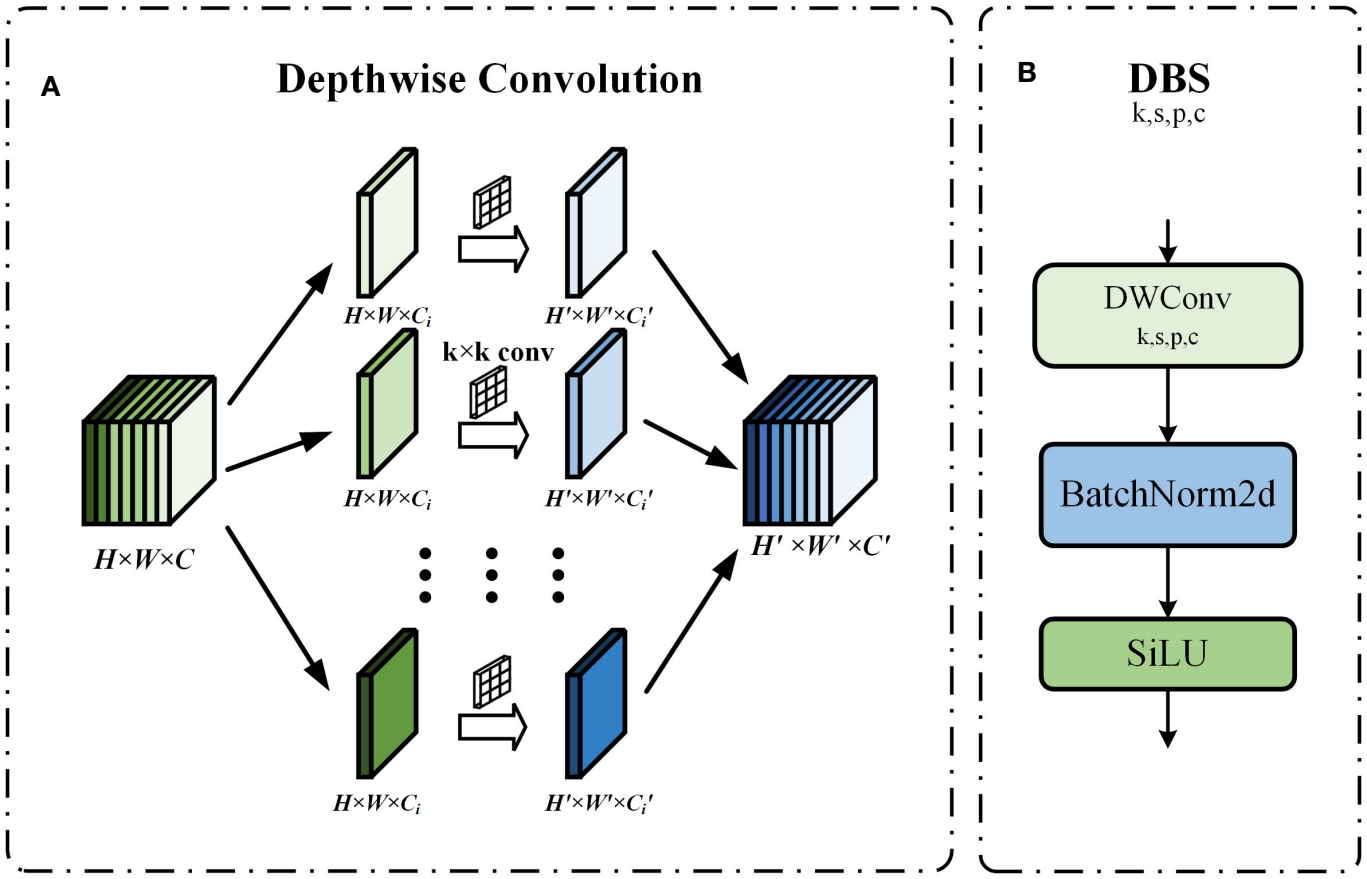
**(A)** Depthwise convolution schematic. **(B)** DBS module structure.


In order to meet the computational efficiency requirements of embedded device deployment and optimize the model structure, we have chosen to convert some CBS modules into DBS modules. The structure of the DBS module is shown in **Figure 7B**, which consisted of depth convolution, batch normalization, and SiLU activation function. Depthwise convolution (DConv) is an efficient variant of traditional 2D convolution. Unlike the traditional 2D convolution which has only one grouping, deep convolution uses the maximum common denominator M of the number of input channels and the number of output channels as the number of groupings to group the feature maps, and each convolution kernel of size k × k performs the feature extraction within the group, and finally the results are stitched together to obtain the output feature maps (Chollet, 2017). The structure of depthwise convolution is shown in **Figure 7A**. Assuming that the input feature map size is H × W × C and the output feature map size is ${\text{H}}^{\prime }\times {\text{W}}^{\prime }\times {\text{C}}^{\prime }$ . The computational quantities of traditional 2D convolution operation and depthwise convolution are shown in Equations (4) and (5), respectively.

(4) $$\text{FLOP}s_{Conv\;=}\;\text{k}\times \text{k}\times \text{C}\times H^{\prime }\times W^{\prime }\times C^{\prime }$$

(5) $$\text{FLOP}s_{DConv\;=}\;\text{k}\times \text{k}\times C_{i}\times H^{\prime }\times W^{\prime }\times C_{i}\prime \times \text{M}$$

By Equation (4-5), since $C_{i}=C/M,\;C_{i}\prime =C^{\prime }/M$ . By calculation, it can be seen that the FLOPs of depthwise convolution is about 1/M of traditional 2D convolution, which significantly reduces the computational burden of the model and decreases the risk of overfitting.

##### 2.3.1.3 PDetect

**Figure 8:**
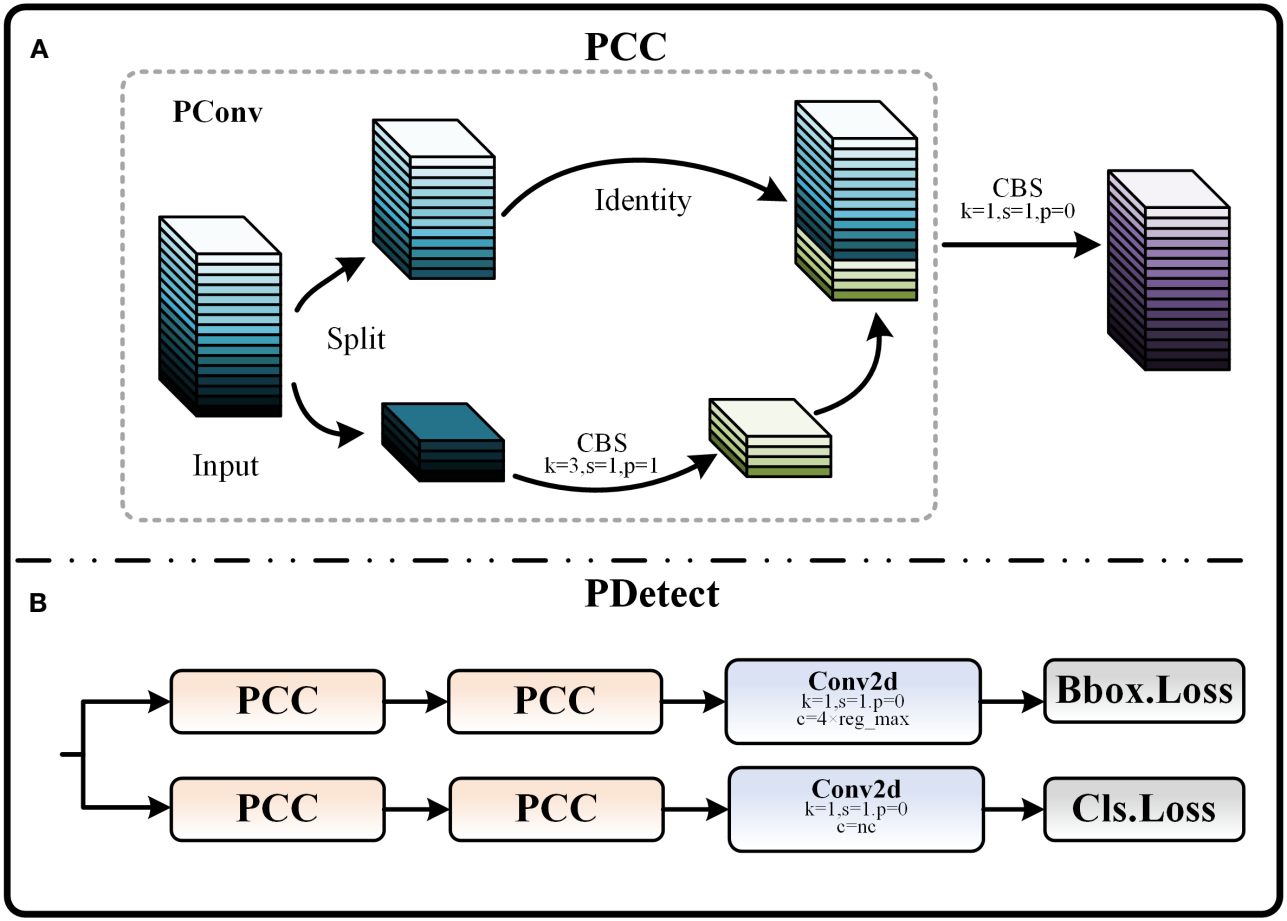
**(A)** Schematic of PCC module. **(B)** PDetect structure.


In order to improve the computational efficiency of the detection head section, we designed the PCC module by combining the ideas of PConv. The structure diagram of the PCC module is shown in **Figure 8A**. PConv is a recently developed lightweight convolutional technique. This technique is able to effectively reduce the number of parameters by reducing redundant computations and memory accesses, and in turn speeds up detection with little or no loss in model accuracy (Chen et al., 2023). The working method of Pconv is as follows: The input feature map X is first divided into two parts X1 and X2 by the ℎr function scaled by r in the channel dimension. Then, X2 is subjected to feature extraction through the CBS module, and the obtained feature map is directly spliced with another part X1 to obtain the output feature map, which significantly reduces the computational complexity of the model. Based on PConv, the PCC module adds a CBS module with a convolutional kernel size of 1×1. This improvement enhances the feature fusion and cross-channel perception of the model without significantly increasing the parameters, thus better enhancing the feature expressiveness of the model. Equations (6) and (7) represent the process by which the PCC module obtains the output feature map Y through a two-part operation, where ℊ stands for feature extraction with a CBS module of convolutional kernel size i, and ⊕ stands for the splicing operation.

(6) $$X_{1},X_{2}=h_{r}(\text{X})\;$$

(7) $$\text{Y}={ℊ}_{1}(X_{1}⊕\;{ℊ}_{3}(X_{2}))$$

Apply the PCC module to the detection head and replace some of the CBS modules to form the PDetect detection head. As shown in **Figure 8B**. At this time, the size of both input and output feature maps are H × W × C . Equation (8) demonstrates the ratio of the FLOPs of the PCC module to that of the traditional convolution operation, which is only 1/5-1/6 of that of the traditional convolution when k = 3, r = 4.


(8) $$\text{s}=\frac{FLOPs_{PCC}}{FLOPs_{Conv}}=\frac{k\;\times \;k\;\times \;C/r\;\times \;W\;\times \;H\;\times \;C/r\;+\;C\;\times \;W\;\times \;H\;\times \;C}{k\;\times \;k\;\times \;C\;\times \;H\;\times \;W\;\times \;C}=\frac{1}{{\text{r}}^{2}}+\frac{1}{{\text{k}}^{2}}$$

#### 2.3.2 Coordinate attention mechanism

**Figure 9:**
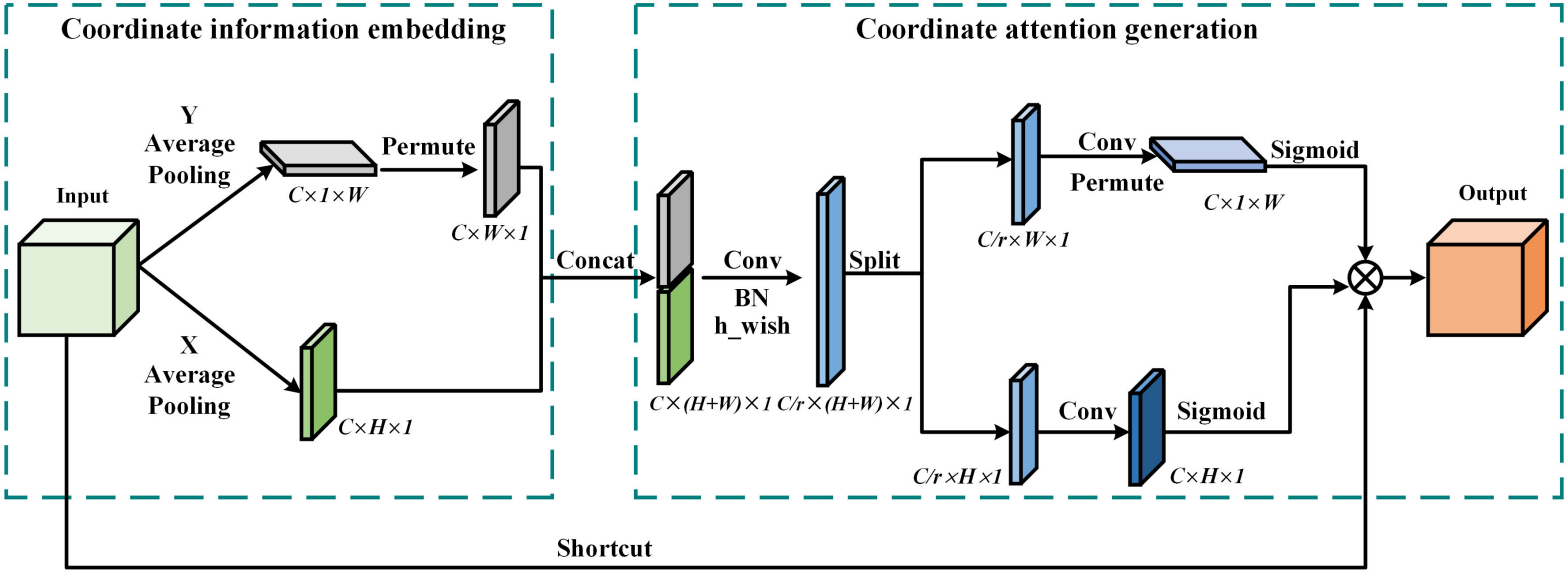
Structure of CA attention mechanism.

In order to enhance the accuracy of the model in locating core regions in complex backgrounds, enhance the attention and feature extraction capabilities of germinating regions, and reduce the impact of lightweight technology on detection accuracy, we introduced the Coordinate Attention (CA) mechanism (Hou et al., 2021). By refining the channel attention into two parallel one-dimensional feature encoding processes and integrating the spatial coordinate information into the generated attention map, CA allows the model to explore and utilize the intrinsic connections between the feature channels in greater depth, thus enhancing the semantic insight of the model and hence the accuracy of the detection. We configure it after the first C2f_Ghost module of the backbone network to realize the attention and extraction of key features dynamically. In addition, the CA mechanism is both flexible and lightweight, and can be easily integrated into lightweight networks, requiring negligible computational overhead. The specific architecture of the CA attention mechanism is demonstrated in **Figure 9**.

CA mechanism can be subdivided into two phases: coordinate information embedding and coordinate attention generation. In the coordinate information embedding stage, first, a channel-by-channel average pooling is performed on the input feature maps, and each channel is encoded using the pooling kernels of (H, 1) and (1, W) for the X and Y axes, respectively. As a result, CA mechanism can capture long-range dependencies within a single channel and retain accurate location information, enhancing the localization capability of the network. In the coordinate attention generation phase, we first concat the previously extracted feature maps in the spatial dimension and then perform a convolution operation for feature dimensionality reduction to reduce the computational burden. After that, Batch normalization and h-swish nonlinear activation operations are performed. The obtained feature map is split into two tensors by spatial dimension, dimensionally expanded by 1×1 convolution, and activated using the sigmoid function to generate a weight matrix with spatial information. Finally, the weight matrix is multiplied with the input feature map to complete the recalibration of the input feature space levels.

#### 2.3.3 Lightweight CARAFE upsampling operator

**Figure 10:**
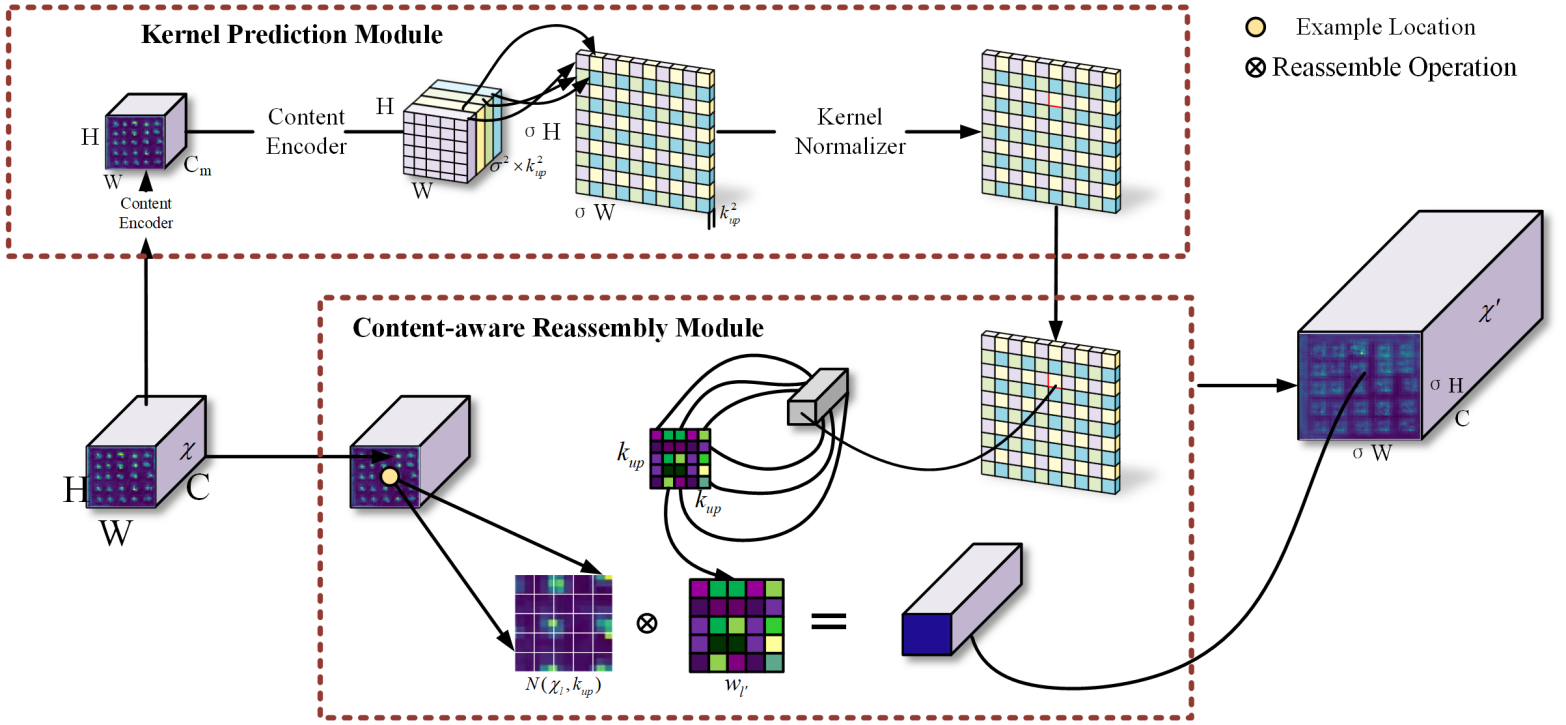
Schematic of the CARAFE upsampling operator.


Feature up-sampling is a key operation in many network architectures, and the quality of the up-sampled generated feature maps is critical to the accuracy of the model. CARAFE has unique content-aware properties that can accurately recover image details while reducing the information loss of small targets such as buds caused by the downsampling process, and does not need to introduce more additional learning parameters (Wang et al., 2019). CARAFE is divided into two main modules, namely, the kernel prediction module and the content-aware reassembly module. The objective is to convert the original feature map X (C × H × W) into the target feature map ${{\text{X}}^{\prime }}^{\;}\left(\text{C}\times \text{σH}\times \text{σW}\right)$ . The positions $l^{\prime }=({\text{i}}^{\prime },{\text{j}}^{\prime })$ on ${\text{X}}^{\prime }$ all correspond to the positions l=(i,j) on X, where $\text{i}=\left\lfloor {\text{i}}^{\prime }/\text{σ}\right\rfloor \text{and}\left\lfloor {\text{j}}^{\prime }/\text{σ}\right\rfloor$ . The up-sampling kernel prediction module mainly predicts the up-sampling kernel $W_{l^{\prime }}$ at each position $l^{\prime }$ of the target feature map through a three-step operation of Channel Compressor, Content Encoder, and Kernel Normalizer. As shown in Equation (9), $\text{N}\;(X_{l},\text{k})$ denotes the k × k adjacent region of the feature map X centered at *l*. The kernel prediction module first compresses the input feature map channels to reduce the subsequent computational load and then converts the number of channels to σ² × k_up_² by convolutional layers with a convolutional kernel size of k_encoder_ × k_encoder,_ where σ is the upsamp ratio. Finally, the channel dimension is expanded in the spatial dimension and normalized with the sigmoid function to form an upsampling kernel of size σH × σW × k_up_². The content-aware reassembly process is shown in equation (10), where r = ⌊k_up_/2⌋. For the target location $l^{\prime }$ , the content-aware reassembly module first finds its corresponding region $\text{N}\;(X_{l},{\text{k}}_{\text{up}})$ on the input feature map, and uses this region to do the dot product with the corresponding location of the up-sampling kernel in order to pay better attention to the information from the correlation points in the localized region, and generates a feature map with better semantic features. The structure of CARAFE is shown in **Figure 10**.

(9) $$W_{l^{\prime }}=\text{ψ}(\text{N}(X_{l},k_{encoder}))$$

(10) $$X_{l^{\prime }}^{\prime }=\sum_{n=-r}^{r}\sum_{m=-r}^{r}W_{l^{\prime }(\text{n,m})}\cdot {\text{X}}_{\left(i+n,j+m\right)}$$

### 2.4 Optimizer improvement

The Adam optimizer combines the main advantages of the adaptive gradient algorithm (AdaGrad) and the root mean square propagation algorithm (RMSProp) have adaptive learning rates, stable convergence, and bias correction mechanisms to avoid models falling into local optimum regions (Kingma and Ba, 2014; Goyal et al., 2017). Thus, it improves the optimization process of the model and enhances the performance of target detection.


(11) $$g_{t}=\nabla_{\theta }f_{t}\left(\text{θ}\right)$$

In Equation (11), $g_{t}$ represents the gradient value, which is the result of taking the partial derivative of the Loss function $\;f_{t}\left(\theta \right)$ with respect to θ .Where t represents the number of iterations.


(12) $$m_{t}={\text{β}}_{1}m_{t-1}+\left(1-{\text{β}}_{1}\right)g_{t}$$

(13) $$v_{t}={\text{β}}_{2}v_{t-1}+\left(1-{\text{β}}_{2}\right)g_{t}^{2}$$

The Adam optimizer uses a separate adaptive learning rate for each parameter, which is obtained by calculating the first-order moments (i.e., the expected value of the gradient) and second-order moments (i.e., the variance of the gradient) of the historical gradient for that parameter. As shown in Equations (12) and (13), m_t_ represents the exponential moving average, considering the gradient momentum of the previous time step. v_t_ represents the squared gradient, considering the squared gradient of the previous time step. β1 and β2 represent the decay rate of the exponentially shifted mean, in this experiment, β1 = 0.937 and β2 = 0.999. This adaptive learning rate mechanism allows the Adam optimizer to automatically adjust the step size of the parameter updates, maintaining good optimization performance in various parameter cases. Where ${\hat{m}}_{t}$ represents the bias-corrected first-order estimation matrix and ${\hat{v}}_{t\;}$ is the bias-corrected second-order estimation matrix.


(14) $${\hat{m}}_{t}=\frac{m_{t}}{1-\beta_{1}^{t}}$$

(15) $${\hat{v}}_{t}=\frac{v_{t}}{1-\beta_{2}^{t}}$$

Adam introduces a bias correction mechanism to prevent too large learning rate due to small estimates of first-order moments and second-order moments of the gradient at the early stage of training. This bias correction allows Adam to maintain a stable learning rate even in the early stages of training. The bias correction process is shown in Equations (14) and (15).


(16) $$\theta_{t+1}=\theta_{t}-\frac{\eta }{\sqrt{{\hat{v}}_{t}}+\epsilon }{\hat{m}}_{t}$$

During each iteration, the parameter update process is shown in Equation (16), where η is the learning rate, and ϵ is a constant parameter.

### 2.5 Evaluation metrics

#### 2.5.1 Model evaluation metrics

To comprehensively evaluate the performance of the pea germination detection model. We considered the accuracy requirements of the model and the lightweight requirements for integrating the model into the embedded devices, the following metrics were selected: precision, recall, average precision (AP), mean average precision (mAP), FLOPs, Params, and Weight Size. Precision, recall, AP, mAP, FLOPs, and Params were calculated as shown in Equations (17)–(22).


(17) $$\text{Precision}=\frac{TP}{TP+FP}$$

(18) $$\text{Recall}=\frac{TP}{TP+FN}$$

(19) $$\text{AP}=\int_{0}^{1}\text{P}(\text{R})\text{dR}$$

(20) $$\text{mAP}=\frac{\int_{\text{q}=1}^{\text{Q}}\text{AP}(\text{q})}{\text{n}}$$

(21) $$\text{FLOPS}=2\times \text{H}\times \text{W}\left(C_{in}K^{2}+1\right)C_{out\;}\;\;$$

(22) $$\text{Params}=C_{in}\times K^{2}\times C_{out\;}\;$$

True positive (TP) is the number of peas whose growth condition was correctly identified by the model, whereas false positive (FP) and false negative (FN) represent the number of peas that were actually present but incorrectly identified and missed by the model, respectively. Accuracy rate is the proportion of all predicted targets correctly identified by the model, and recall rate is the proportion of all actual targets correctly identified by the model. On the basis of these two rates, we plotted precision–recall (PR) curves for each category. The area under the curve is the AP value for that category, with a value close to 1 indicating good model performance. The mAP is the mean value of the multi-category AP, which is a common evaluation metric in target detection and intuitively reflects the current model performance. Model computational complexity is reflected by FLOPs, model lightweighting is assessed by the number of Params, and Weight Size can be used to measure the ease of integration of the model and its suitability for implantation in lightweight devices. Our goal is to achieve maximum lightweighting and ease of integration while maintaining model accuracy.

#### 2.5.2 Evaluation metrics of seed germination vigor

The quality and agricultural value of pea seeds are mainly assessed by two key indicators: germination rate and germination index. Germination rate refers to the ratio of the number of normally germinated seeds to the total number of seeds in a given environment, which directly reflects seed vitality and germination potential and is the basic index for seed quality assessment. The germination index shows the overall activity of the seed by measuring the number and rate of germination of seeds within a certain period. High germination index represents seeds with strong vitality and growth potential. The germination index is critical in the pursuit of maximum yield agricultural production. These two quantitative parameters are scientific and objective and can accurately reflect the seed viability of seeds under specific environmental conditions and screen for quality peas with excellent genotypes (Okçu et al., 2005). The formulae for germination rate and germination index are shown in Equations (23) and (24). “Nt”,”N”, “Gt” and “Dt” represent the number of seeds germinated on “t” days, the total number of seeds tested, the number of seeds germinated on “t” days, and the seed growth time, respectively.


(23) $$\text{Germination rate}=\frac{N_{t}}{N}\times 100\%$$

(24) $$\text{Germination index}=\sum (\frac{G_{t}}{D_{t}})$$

## 3 Results and discussion

### 3.1 Training environment and hyperparameter settings

In this experiment, the experimental environment was configured as follows. We used an Intel(R) Xeon(R) Gold 6248R @ 3.00GHz processor with an NVIDIA GeForce RTX3090 graphics card. The deep learning model framework used Pytorch 2.0.0 and Python 3.8, CUDA version was selected as 11.7, and the operating system was selected as Windows 11. We randomly divided the dataset into training, validation, and testing sets by 3:1:1. To ensure fairness and comparability of model effects, we did not use pre-training weights for the various model training processes in all ablation experiments and comparison experiments. We resized the input image size to 640 × 640, the number of iterations to 100. Some of the important hyperparameter settings of the model in the training phase are shown in **Table 1**.

**Table 1:** Model hyperparameter settings.

Parameters	setup
Epoch	100
Batch size	8
NMS IoU	0.65
Image Size	640×640
Initial Learning Rate	1×10^-2^
Final Learning Rate	1×10^-4^
Momentum	0.937
Weight-Decay	1×10^-4^

### 3.2 CARAFE operator performance evaluation

The two core parameters of the CARAFE upsampling operator, k_encoder_ and k_up_, denote the encoder kernel size and the upsampling kernel size, respectively. k_encoder_ is responsible for controlling the efficiency and accuracy of feature encoding, whereas k_up_ affects the reconstruction ability of detailed features. The moderate choice of parameters is crucial,.too large or too small parameters may adversely affect the accuracy, computational efficiency, and lightweight characteristics of the model.

We conducted a series of experiments to explore the effects of different k_encoder_ and k_up_ values on the detection results of the YOLOv8-Peas model (all other training parameters are kept the same), in order to find the optimal parameter configurations to improve the model performance. The experimental results are shown in **Table 2**. By analyzing the tabular data, when k_up_ is small, although the number of model parameters is small, the reconstruction ability of the upsampling operator is insufficient to recover detailed features, which leads to a decrease in the accuracy of the model. When k_encoder_ is small, the encoder perceptual field is limited and sufficient contextual information cannot be obtained, which affects the accuracy of the model. The oversized k_up_ and k_encoder_ not only increase Params significantly but also degrade the model performance. Comparing the experimental results, we believe that k_up_ of 3 and k_encoder_ of 5 are the optimal parameter configurations for the sampling operator on CARAFE. This parameter combination ensures the highest accuracy of the model while effectively controlling the model complexity and the number of Params.

**Table 2:** Detection results for different k_up_ and k_encoder_ values.

k_up_	k_encoder_	mAP0.5(%)	Params(M)	FLOPs(G)	Weight Size(MB)
1	3	98.5	**1.06**	**3.0**	**2.4**
1	5	98.4	1.07	3.0	2.5
3	3	98.5	1.10	3.1	2.5
3	5	**98.7**	1.17	3.2	2.7
5	5	98.6	1.38	3.6	3.1
5	7	98.5	1.68	4.2	3.7
7	7	98.5	2.29	5.4	4.9

Bold indicates the best experimental results.

**Figure 11:**
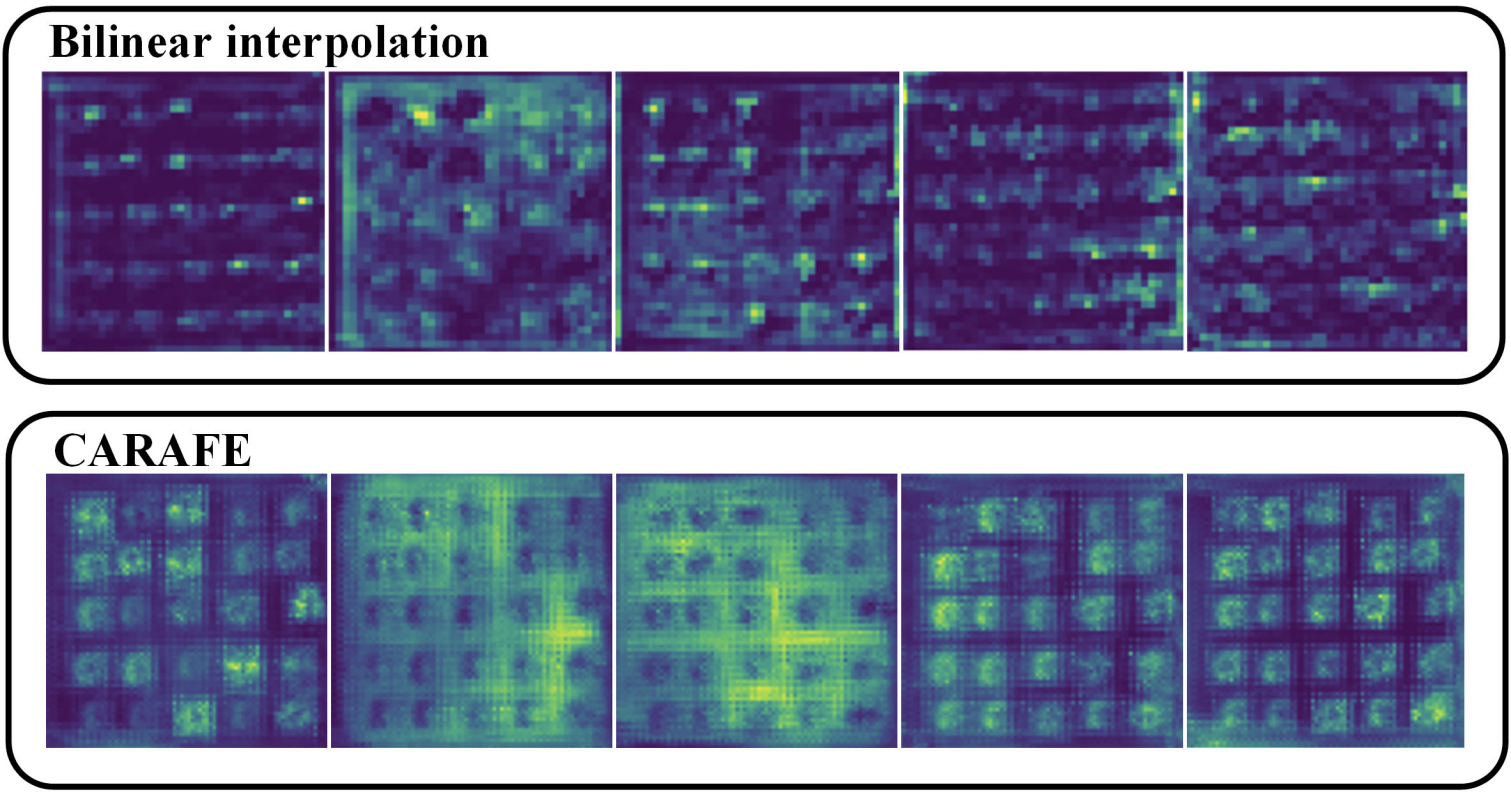
Comparison of bilinear interpolation and CARAFE upsampling feature maps.

To demonstrate the significant superiority of the CARAFE upsampling operator intuitively, we compared and analyzed the feature maps obtained after using the bilinear interpolation method and the CARAFE upsampling operator. As shown in the **Figure 11**, the upsampled feature maps of the bilinear interpolation method exhibited stark differences from those of CARAFE after upsampling. The feature maps generated by the CARAFE operator exhibited significant advantages in terms of accuracy and detailed feature retention, providing clear, fine, and informative feature maps. These results highlight the unique advantages of the CARAFE upsampling operator in feature recovery and information reconstruction, further justifying our use of the CARAFE upsampling operator.

### 3.3 Ablation experiments

In the ablation experiments, we evaluated the role of each module in detail. The experimental results are shown in **Tables 3** and **4**. First, introducing the C2f-Ghost module into YOLOv8-n reduced the Params, FLOPs, and Weight Size by 28.9%, 24.3%, and 25.8%, respectively, which effectively reduces the model size and computation cost, and improves the efficiency of feature extraction. After optimizing part of the CBS structure into DBS, the number of Params, FLOPs, and Weight Size were further reduced by 26.8%, 16.2%, and 26%, respectively, and the FPS improved by 3.9. With the addition of the CARAFE upsampling operator, the model achieved an improvement in accuracy due to the effectiveness of CARAFE in recovering image details, despite the slight increase in the number of Params and FLOPs. The addition of the PDetect detection header effectively reduces redundant computations and memory accesses, resulted in a significant reduction of 31.9%, 41.8%, and 28.9% in the number of Params, FLOPs, and Weight Size, respectively, and a 5.6 increase in FPS. Then the CA module was added to the backbone network part, which improved the model’s ability to localize and semantically understand the germination area, and reduced the influence of the model’s feature extraction due to the overlapping of the root system, and the model’s mAP was improved to 98.4. Finally we replaced the optimizer with Adam, and the mAP further improved to 98.7%, which was in line with YOLOv8-n. We designed YOLOv8-Peas with 61%, 61.2%, and 56.5% reduction in the Params, FLOPs, and Weight Size, respectively, compared with the benchmark model. Thus, we effectively reduced the complexity and optimizing the efficiency of the model, making it suitable for low-performance devices and easy to integrate. The FPS reached 116.2, which was better than that of the benchmark model, but it met the demand for real-time detection of pea sprouting. The ablation experiments clearly demonstrated the superiority and effectiveness of our model in terms of accuracy and light weight, proving the feasibility of our designed scheme applied to pea seed germination detection.

**Table 3:** Results of ablation experiments in terms of model lightweighting.

Model	mAP@0.5 (%)	Params (M)	FLOPs (G)	Weight Size (MB)	FPS
YOLOv8-n	98.7	3.011	8.2	6.2	**151.5**
C2fGhost	98.5	2.145	6.2	4.6	136.9
C2f-Ghost+DBS	98.3	1.580	5.2	3.4	140.8
C2f-Ghost+DBS+CARAFE	98.5	1.710	5.5	3.7	125
C2f-Ghost+DBS+CARAFE+PDetect	98.2	**1.171**	**3.2**	**2.7**	130.6
C2f-Ghost+DBS+CARAFE+PDetect +CA	98.4	1.176	**3.2**	**2.7**	116.2
YOLOv8-Peas	**98.7**	1.176	**3.2**	**2.7**	116.2

Bold indicates the best experimental results.

**Table 4:** Results of ablation experiments in terms of model detection accuracy.

Model	mAP@0.5 (%)	AP_sprout_ (%)	AP_not sprout_ (%)	Precision	Recall
YOLOv8-n	**98.7**	98.6	**98.7**	97.6	97.0
C2fGhost	98.5	98.4	98.5	97.3	**97.1**
C2f-Ghost+DBS	98.3	98.1	98.6	97.3	96.8
C2f-Ghost+DBS+CARAFE	98.5	98.5	98.4	97.0	96.7
C2f-Ghost+DBS+CARAFE+PDetect	98.2	98.0	98.3	97.0	96.5
C2f-Ghost+DBS+CARAFE+PDetect +CA	98.4	98.2	98.6	96.9	96.8
YOLOv8-Peas	**98.7**	**98.8**	98.5	**97.8**	96.9

Bold indicates the best experimental results.

### 3.4 Comparison experiments

As shown in the **Table 5**, the detection accuracy and various metrics of YOLOv8-Peas were compared with the lightweight versions of YOLOv3 to YOLOv8, as well as the classical model Deformable–DETR in the transformer architecture(Zhu et al., 2020; Ge et al., 2021; Xu et al., 2022). The results of each indicator for different models are shown in **Table 5**. The mAP of the model, the recognition accuracy of sprout and not sprout peas (AP_sprout_ and AP_not sprout_), Params, FLOPs, and Weight Size were mainly examined. In terms of the computational complexity of the model, YOLOv8-Peas was much lower than all the other models in terms of the Params, FLOPs, and Weight Size, which were only 1.17M, 3.2G, and 2.7MB, respectively, with 80.5%, 75.7%, and 78% decreases compared with YOLOv7-Tiny, a network commonly used for target detection in agriculture. Compared with YOLOv5-s, it decreased by 83.3%, 80%, and 81.2%,respectively. In terms of detection accuracy, YOLOv8-Peas achieved 98.7% on mAP, reaching the highest level, which is 1.7%, 1.0%, and 0.3% higher than YOLOv5-s, YOLOv6-n, and YOLOv7-Tiny, respectively. It achieved 98.8% on AP_sprout_, surpassing all other models. Reaching 98.5% on AP_not sprout_, which is in the leading position. It can effectively identify the two growth states of peas.

**Table 5:** Results of each indicator for different models.

Model	mAP@0.5 (%)	AP_sprout_ (%)	AP_not sprout_ (%)	Params (M)	FLOPs (G)	Weight Size (MB)
Deformable-DETR	98.2	97.9	98.5	39.84	176.83	457.66
YOLOv3	90.6	86.0	95.3	61.52	155.2	235.07
YOLOv4-tiny	81.2	74.4	87.9	5.89	16.17	22.48
YOLOv5-s	97.0	96.6	97.5	7.02	16.0	14.4
YOLOv6-n	97.7	97.5	97.9	6.43	11.34	10.2
YOLOv7-tiny	98.4	98.2	98.6	6.01	13.2	12.3
YOLOv8-n	**98.7**	98.6	**98.7**	3.01	8.2	6.2
YOLOX-tiny	98.2	98.0	98.5	5.03	15.23	19.44
DAMO-YOLO-T	97.8	97.5	98.1	8.5	18.1	31.1
YOLOv8-Peas	**98.7**	**98.8**	98.5	**1.17**	**3.2**	**2.7**

Bold indicates the best experimental results.

Comparative experiments demonstrated that our model not only has excellent accuracy but also a clear advantage in computational complexity for the pea seed germination detection task. Thus, YOLOv8-Peas is particularly suitable for scenarios where resources are limited but high accuracy detection is required, such as real-time pea germination detection on embedded devices or mobile devices.

**Figure 12:**
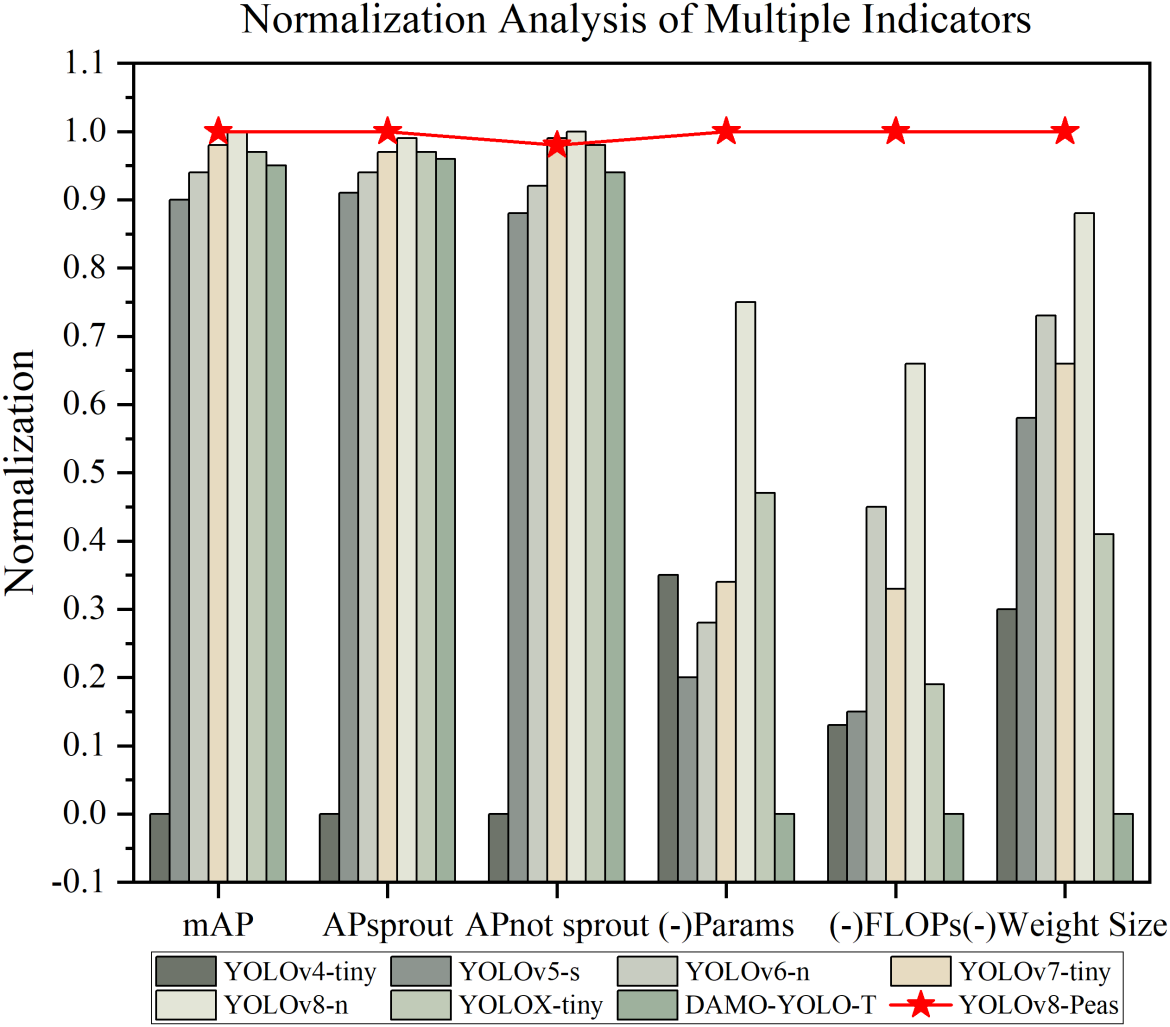
Multi-indicator normalized analysis.

To demonstrate the performance of the models in these evaluation metrics more intuitively, we plotted the normalized histograms of the YOLOv4-Tiny,YOLOv5-s,YOLOv6-n, YOLOv7-Tiny,YOLOv8-n, and YOLOv8-Pea models. The values were obtained by the Min-Max standard normalization process. The Params, FLOPs, and Weight Size were processed in the opposite way; the larger the true value, the lower the score. The closer the value is to 1, the better the model performs on this metric. The results are shown in **Figure 12**. Star-tagged YOLOv8-Peas achieved the highest level in the five indexes of Params, FLOPs, Weight Size, mAP, and AP_sprout_, and it was far ahead of other models in Params, FLOPs, and Weight Size. The overall performance has reached the best, with high precision and lightweight characteristics, which can better meet the actual needs of agricultural production.

### 3.5 Predicting performance

**Figure 13:**
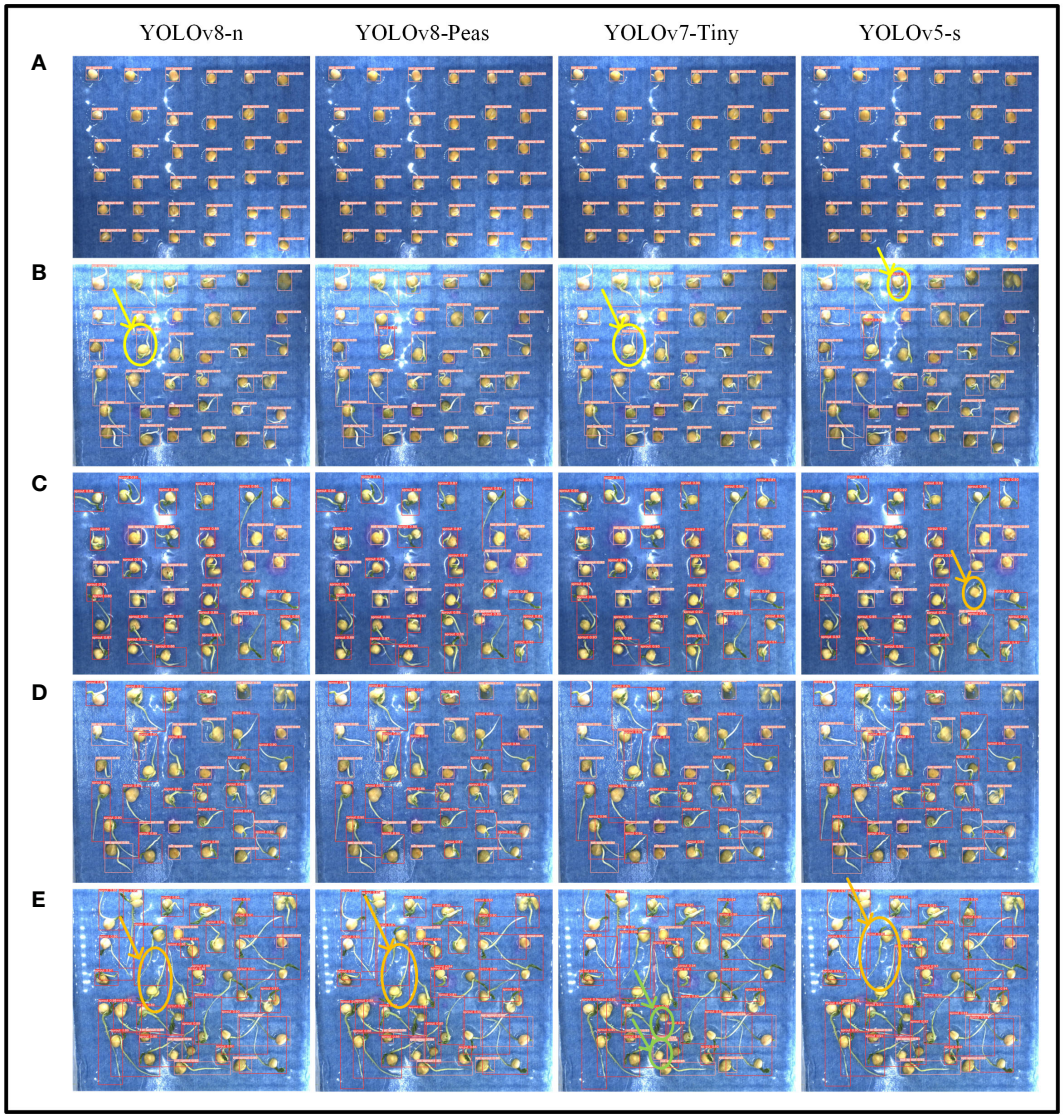
Detection results of four lightweight algorithms for different growth conditions of peas. **(A–E)** Five different stages of pea germination.

We compared and analyzed the recognition effect of YOLOv8-Peas with three lightweight models, YOLOv8-n, YOLOv7-Tiny, and YOLOv5-s, at various stages of pea germination, and the detection results are shown in **Figure 13**. The pink marking area indicates the identified ungerminated peas, while the red marking corresponds to the germinated peas. In order to clearly demonstrate the problems in detection, we have circled the areas where the model misdetects in yellow, the areas where it misses detection in orange, and the areas where it repeats detection in green. In stage (b), which is in the early stage of sprouting, the pea shoot length gradually reaches the sprouting standard, and the models YOLOv8-n, YOLOv5-s, and YOLOv7-Tiny are all misdetected, but YOLOv8-Peas accomplished the detection well. At stage C, reaching the middle stage of seed germination, and most of the peas have sprouted, YOLOv5-s showed a missed detection. At stage E, the late stage of pea seed germination was reached and the root system interlacing condition was severe, with a small number of missed detections for YOLOv8-n, YOLOv8-Peas, and YOLOv5-s. YOLOv7-Tiny showed a problem of duplicate detection for individual peas. Overall, YOLOv8-Peas improved in detection performance compared with YOLOv5-s and YOLOv7-Tiny, with slightly higher accuracy than YOLOv8-n. It can effectively complete the detection task in the environment of intertwined and shaded roots, and realize the efficient detection of pea germination, which is helpful for the screening of new pea varieties with drought resistance.

### 3.6 Detection of drought tolerance sprouting vigor in peas

The dependence of the seed germination stage on water conditions is particularly sensitive, and drought stress leads to changes in many physiological and biochemical processes that control plant growth and productivity, causing severe impacts on pea growth and development and resulting in reduced yield and quality (Daie and Patrick, 1988). Therefore, we subjected different genotypes of pea seeds to drought stress at the germination stage to compare their drought resistance.

**Figure 14:**
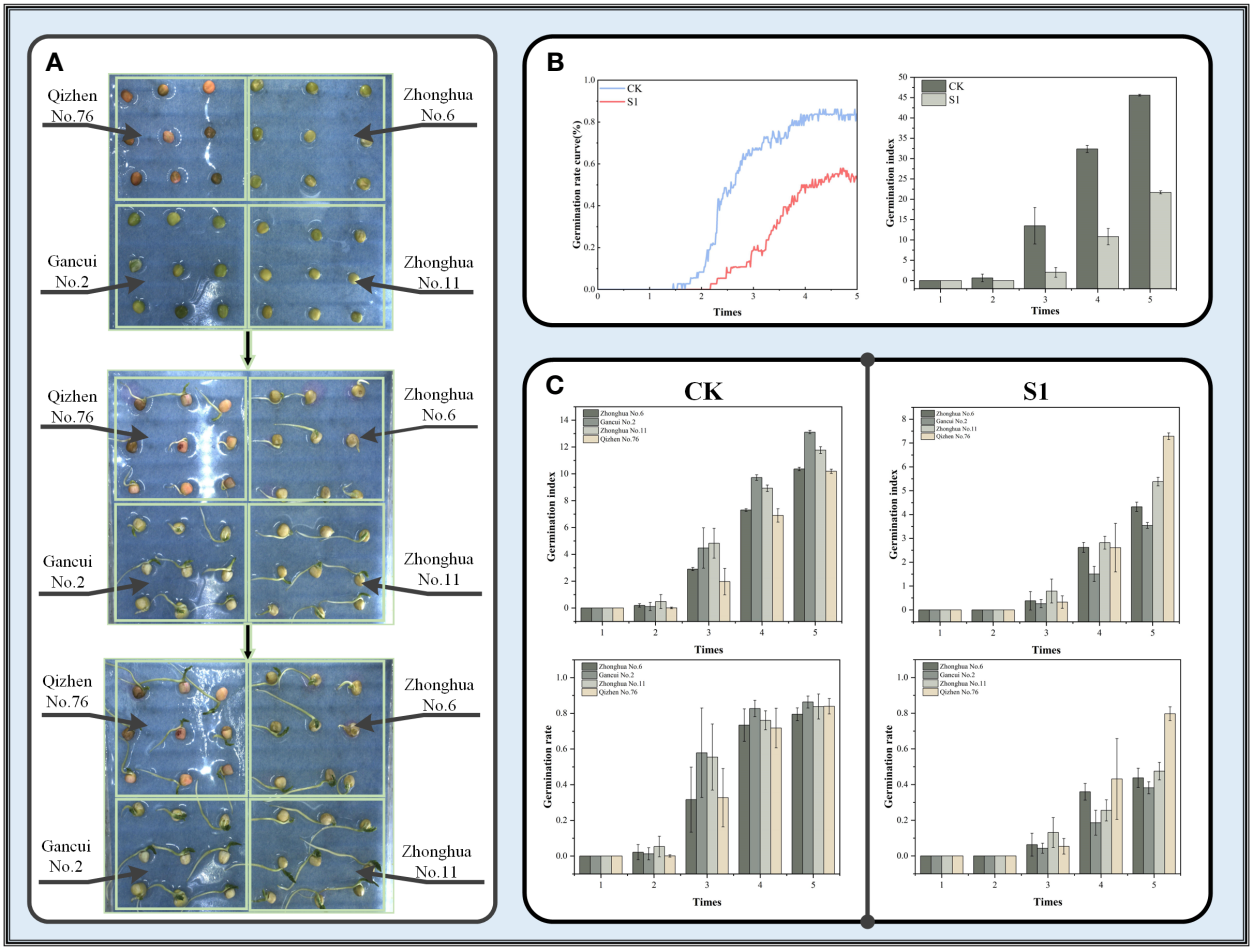
**(A)** Schematic of the placement and growth process of different genotypes of peas. **(B)** Comparison of germination rate and germination index under two conditions. **(C)** Results of germination rate and germination index of four cultivars of peas under two conditions with time.

Images from the drought stress dataset were examined using YOLOv8-Peas, and germination rate and germination index were used as indicators to assess the germination vigor of four genotypes of peas under different drought conditions. A schematic of seed placement and a diagram of the growth process are shown in **Figure 14A**. As shown in **Figure 14B**, the drought environment simulated by 10% of PEG6000 solution had a significant effect on the germination rate of peas, resulting in an approximate 31.1% decrease in cumulative germination rate and an approximate 52.4% decrease in germination index. **Figure 14C** shows the germination rates and germination index of these four pea genotypes over time in both environments. In the control (CK) environment, pea seeds started to germinate from the second day and reached about 80% germination rate by the fifth day. However, the germination speed of pea seeds was significantly depressed under drought conditions, with all peas starting to germinate from the third day. This response was more pronounced in Zhonghua No. 6 and Zhonghua No. 11 peas, which had less than 50% germination and germination index of 4.3 and 5.3, respectively, indicating a significant decrease compared with the control group. Gancui No.2 was the most sensitive to the effects of drought, and its germination rate on the fifth day was only 38%, with a germination index of only 3.5, showing poor drought resistance. By contrast, Qizhen No.76 was more tolerant to drought conditions and showed no significant changes in the germination rate and germination index relative to the control group. Although a slight decrease in the germination speed was found at middle stage of seed germination, the germination rate could still reach about 80% at the final stage with a germination index of 7.3. These results experimentally confirmed that all four pea cultivars were affected by drought conditions in the middle of germination. Meanwhile, Qizhen No. 76 showed high drought tolerance and could be grown in areas with scarce water or drought.

## 4 Summary, limitations and future work

In order to achieve full-time non-destructive detection of pea seed germination vigor and in-depth analysis of the effects of drought stress on various stages of pea germination, so as to screen out pea varieties with excellent drought resistance, and also need to take into account the reduction of the cost of the deployment of the model in order to enhance the potential of its practical application. To address this need, this paper proposes the lightweight pea germination detection model YOLOv8-Peas. First, we use a variety of simple and effective data enhancement techniques to provide data in a variety of specific scenarios, to solve the problems of difficult data collection and high cost of human labeling, and to improve the detection results of the model under complex environmental changes. Secondly, in order to enhance the practical value of the model and meet the demand of embedded deployment. In this paper, C2f-Ghost is constructed to reduce the number of parameters and computational complexity, and improve the efficiency of feature extraction. And the DBS module is used to replace part of the CBS module to achieve effective lightweighting while maintaining the model performance, which brings substantial benefits to the overall optimization and deployment. Meanwhile, the PCC module is used to optimize the detection head to reduce redundant computation and improve the detection speed. Finally, to solve the problem of poor feature extraction for small targets such as buds. We add CA attention mechanism in the trunk part to enhance the model’s ability to recognize and localize the key sprouting regions in the complex background. And the CARAFE operator is used in the up-sampling process to enhance the model’s ability to perceive details, reduce the information loss of small targets caused in the down-sampling process, and improve the detection accuracy.

The experimental results show that YOLOv8 Peas achieved 98.7% in mAP, with a decrease of 61.2%, 61%, and 56.5% in Params, FLOPs, and Weight Size compared to YOLOv8-n. They were only 1.17M, 3.2G, and 2.7MB, achieving a detection speed of 116.2FPS. Compared with other lightweight detection models in the YOLO series, YOLOv8-Peas offers a smaller model size and better detection performance. The method considers detection accuracy, computational complexity, and weight file size, making it suitable for deployment on low-cost devices and mobile terminals.

To demonstrate the practical application of the model, we simulated drought environments by inhibiting water uptake by peas using PEG6000 solution, and tested the germination rate and germination index of four genotypes of peas in two different drought environments. Histograms reflecting the germination rate and germination index of pea seeds over time were plotted to compare and analyzed the differences in germination speed and seed vigor of peas under different drought conditions. The pea seed with the best drought tolerance, Qizhen No.76, was selected, and this research result provides insight into drought breeding technology for peas.

However, the present method has some limitations. Misdetection occurs when pea buds reach a length near the germination criterion. It may be due to the fact that the model is not sensitive to length information, and the model cannot be trained to understand well the criteria for judging germination in terms of length. Subsequently, techniques such as semantic segmentation and key point detection can be considered to be combined with object detection to help the model improve its ability to perceive the length. Subsequently, techniques such as semantic segmentation and key point detection can be considered to be combined with object detection to help the model improve its ability to understand the length.

In the future, we will further develop our detection model to extract other characteristics of pea seed germination such as roundness, area, shoot length and other parameters, combine with germination rate and germination index to conduct a full time-series analysis of pea growth status with multiple indicators, analyze the effect of drought on the stage of pea germination in more detail, and deploy our model and system to embedded devices. We hope that our proposed work can help users achieve low-cost and high-accuracy screening of high-quality genotypes of pea seeds.

## Data availability statement

The raw data supporting the conclusions of this article will be made available by the authors, without undue reservation.

## Author contributions

HJ: Methodology, Software, Validation, Writing – original draft. FH: Data curation, Investigation, Software, Writing – review & editing. XF: Formal Analysis, Project administration, Supervision, Writing – review & editing. CC: Formal Analysis, Methodology, Supervision, Writing – review & editing. CW: Data curation, Supervision, Writing – review & editing. LT: Data curation, Formal Analysis, Writing – review & editing. YS: Writing – review & editing, Data curation, Supervision.
