# Supplementary material for: YOLOv8-Peas: a lightweight drought tolerance method for peas based on seed germination vigor
Source: Front Plant Sci. 2023 Sep 28;14:1257947. doi: 10.3389/fpls.2023.1257947 (PMC10568755; doi:10.3389/fpls.2023.1257947)
Supplement: Supplementary file 1 [file DataSheet_1.pdf]

Supplementary Material

1 Supplementary Figures and Tables

1.1 Detailed traits of 4 genotypes of peas

We collected data on the traits of four genotypes of peas, organized in the table below, with detailed descriptions of each genotype.

	parameters	Zhonghua No.6	Zhonghua No.11	Qizhen No.76	Gancui No.2
growth parameter	Height	40-50	40-50	160-200	50
	Single plant pods	7-10	8-15	10-12	7-10
	Pod length	7-9	7-9	7-8	7
	Pod width	1.2	1.4	1.2	1.2
	Single pod	6-8	8-9	7-9	6
growing period	spring sowing areas	65-75	65-75	/	/
	winter cropping area	90-100	90-100	/	/
	overwintering of seedlings	150	150	/	/

Supplementary Table 1 Detailed traits of 4 genotypes of peas.

Zhonghua No.6:The stem and leaves are dark green. The plant produces white flowers and hard pods. The internodes are swollen. In the early stage, the green pods yield high output, accounting for about 50% of the total production. The pods are large and full, making them more popular compared to the smaller pods. The dried peas are light green with a hundred-grain weight of approximately 25 grams. The plant has strong adaptability, cold tolerance, and resistance to powdery mildew.

Zhonghua No.11:The stem and leaves are of a deep green hue. The plant produces white flowers with firm pods. The internodes exhibit swollen granules. During the early growth stages, the yield of green pods is high, accounting for approximately 50% of the total production. The pods are large and plump. The dried peas are a pale green color with a hundred-grain weight of around 26 grams. Both immature fresh green pea pods and green pea grains are light green in color, with the hundred-grain weight of the green peas being approximately 42 grams.

Qizhen No.76:The sweet and crispy pea belongs to vining varieties and is early-maturing. The plant height ranges from 160 to 200 centimeters. It possesses smaller stems and leaves with strong branching characteristics. The flowers are white in color. The tender pods mature in approximately 60-70 days, exhibiting a sweet and crisp texture with a sugar content of 13.5°Brix. The pod shape is medium-sized and uniform, with an average pod length of about 8 centimeters. The color of the pod is a jade-like green, with a slightly rounded appearance. The variety demonstrates moderate resistance to powdery mildew and rust diseases, and it also possesses strong cold tolerance. In its first growth cycle, the yield per mu is 1600 kg, showing a 23% increase in production compared to the American sweet pea reference. In its second growth cycle, the yield per mu is 1680 kg, which is a 24.4% increase in production compared to the American sweet pea reference.

Gancui No.2:The plant stands at approximately 50 centimeters in height, with light green stems and leaves. It produces white flowers and firm pods. The peas are large-grained, offering a crisp and sweet taste. Typically, a single plant bears 7-10 pods, though some may produce more than 10. The average pod length is about 7 centimeters, with a width of approximately 1.2 centimeters, and contains around 6 grains per pod. The first pod forms quite low on the plant, situated about 5-10 centimeters from the base.

1.2 Vigor of pea germination under different drought conditions

Germination days	Varieties	Treatment	Germination rate	Germination index
3	*Zhonghua No.6*	CK	31.63±18.22	2.89±0.12
		S1	6.32±6.41	0.37±0.38
	Zhonghua No.11	CK	55.47±18.54	4.82±1.11
		S1	13.11±8.35	0.78±0.50
	Qizhen No.76	CK	32.71±16.32	1.96±0.97
		S1	5.40±4.37	0.32±0.26
	Gancui No.2	CK	57.87±25.08	4.47±1.50
		S1	4.32±2.83	0.26±0.16
4	*Zhonghua No.6*	CK	73.38±9.08	7.30±0.09
		S1	35.95±4.66	2.61±0.20
	Zhonghua No.11	CK	76.08±5.32	8.92±0.23
		S1	25.54±5.94	2.81±0.26
	Qizhen No.76	CK	71.75±11.10	6.89±0.49
		S1	43.13±22.63	2.60±1.01
	Gancui No.2	CK	82.71±4.62	9.72±0.20
		S1	18.59±6.98	1.50±0.31
5	*Zhonghua No.6*	CK	79.47±3.56	10.36±0.12
		S1	43.75±5.35	4.32±0.19
	Zhonghua No.11	CK	83.79±7.02	11.76±0.25
		S1	47.54±4.92	5.37±0.17
	Qizhen No.76	CK	83.95±4.32	10.18±0.15
		S1	79.70±3.86	7.28±0.14
	Gancui No.2	CK	86.34±3.35	13.10±0.12
		S1	38.19±3.35	3.54±0.12

Supplementary Table 2 Germination vigor of four genotypes of pea under different drought conditions

1.3 experiment hyperparameter settings

In order to ensure the repeatability and rigor of the experiment, we have provided detailed training parameters for the model in the supplementary materials for readers to use.

Parameters	setup
Epoch	100
Batch size	8
NMS IoU	0.65
Image Size	640×640
Initial Learning Rate	1×10^{-2}
Final Learning Rate	1×10^{-4}
Momentum	0.937
Weight-Decay	1×10^{-4}
Close-mosaic	10
Warmup-epochs	3
Warmup-momentum	0.8
Warmup-bias-lr	0.1
box	7.5
cls	0.5
dfl	1.5
Hsv_h	0.015
Hsv_s	0.7
Hsv-v	0.4
fliplr	0.5

mosaic	1.0

Supplementary Table 3 Detailed training parameters.
